# The Effect of Paracetamol on Core Body Temperature in Acute Traumatic Brain Injury: A Randomised, Controlled Clinical Trial

**DOI:** 10.1371/journal.pone.0144740

**Published:** 2015-12-17

**Authors:** Manoj K. Saxena, Colman Taylor, Laurent Billot, Severine Bompoint, John Gowardman, Jason A. Roberts, Jeffery Lipman, John Myburgh

**Affiliations:** 1 Division of Critical Care and Trauma Division, The George Institute for Global Health, Sydney, New South Wales, Australia; 2 Department of Intensive Care Medicine, St. George Hospital Clinical School, University of New South Wales, New South Wales, Australia; 3 University of Sydney, Sydney Medical School, New South Wales, Australia; 4 Statistics Division, The George Institute for Global Health, Sydney, New South Wales, Australia; 5 Department of Intensive Care Medicine, Royal Brisbane and Women’s Hospital, Brisbane, Australia; 6 Burns, Trauma and Critical Care Research Centre, The University of Queensland, Brisbane, Australia; 7 Faculty of Health, Queensland University of Technology, Brisbane, Australia; Erasmus Medical Centre, NETHERLANDS

## Abstract

**Background:**

Strategies to prevent pyrexia in patients with acute neurological injury may reduce secondary neuronal damage. The aim of this study was to determine the safety and efficacy of the routine administration of 6 grams/day of intravenous paracetamol in reducing body temperature following severe traumatic brain injury, compared to placebo.

**Methods:**

A multicentre, randomised, blind, placebo-controlled clinical trial in adult patients with traumatic brain injury (TBI). Patients were randomised to receive an intravenous infusion of either 1g of paracetamol or 0.9% sodium chloride (saline) every 4 hours for 72 hours. The primary outcome was the mean difference in core temperature during the study intervention period.

**Results:**

Forty-one patients were included in this study: 21 were allocated to paracetamol and 20 to saline. The median (interquartile range) number of doses of study drug was 18 (17–18) in the paracetamol group and 18 (16–18) in the saline group (P = 0.85). From randomisation until 4 hours after the last dose of study treatment, there were 2798 temperature measurements (median 73 [67–76] per patient). The mean ± standard deviation temperature was 37.4±0.5°C in the paracetamol group and 37.7±0.4°C in the saline group (absolute difference -0.3°C; 95% confidence interval -0.6 to 0.0; P = 0.09). There were no significant differences in the use of physical cooling, or episodes of hypotension or hepatic abnormalities, between the two groups.

**Conclusion:**

The routine administration of 6g/day of intravenous paracetamol did not significantly reduce core body temperature in patients with TBI.

**Trial Registration:**

Australian New Zealand Clinical Trials Registry ACTRN12609000444280

## Introduction

Pyrexia, defined as temperature greater than 37°C[1–5], is common in patients with traumatic brain injury (TBI) during the initial 72 hours following admission to the intensive care unit (ICU)[6–10]. Pharmacological agents and physical interventions are commonly used to reduce pyrexia in these patients[6, 7].

The rationale for reducing pyrexia is based on evidence from animal models of TBI that suggest that normothermic conditions may be associated with improved histopathological and neurobehavioural outcomes compared to hyperthermic conditions[11, 12]; from observational studies that report that patients with TBI who develop pyrexia have a higher incidence of adverse outcomes[8, 10, 13] compared to patients who do not develop pyrexia, and from clinical studies that report that reduction of temperature may be associated with a reduction in intracranial pressure[14–16]. There is uncertainty whether reducing or preventing pyrexia is associated with improved patient-centred outcomes[17–21].

Paracetamol is widely used as an early antipyretic pharmacological agent after TBI[6, 7, 22, 23]. The mechanism of the anti-pyretic action of paracetamol is based on inhibition of prostaglandin synthesis within the central nervous system[24], including indirect actions of paracetamol metabolites on cannabinoid[25] and vanilloid[26] receptors. Despite this common practice, there is a paucity of high-quality evidence evaluating its efficacy and safety in this patient population[17].

A previous phase II, randomised, placebo-controlled trial of paracetamol administered by the enteral route in patients with acute ischaemic stroke reported that while 3g/day of paracetamol did not reduce body temperature, 6g/day of paracetamol reduced body temperature by 0.3°C compared to placebo[27], without adverse effects. These observations were confirmed in a subsequent phase III clinical trial that enrolled 1400 participants in a similar population[2].

Based on these data, we sought to determine the efficacy and safety of the early, routine administration of 6g/day of intravenous paracetamol in reducing core body temperature in patients with severe TBI compared to placebo.

## Methods

### Study oversight

We conducted an investigator-initiated, two-centre, parallel-group, blind, randomised, placebo-controlled trial. The Human Research and Ethics Committee of South Western Sydney Area Health Service granted ethical approval for the study (approval number: 09/NEPEAN/43: 14/10/2009); prospective written consent from the patient’s substitute decision maker was required and we obtained prospective written consent for all patients.

### Participants and study setting

Patients with an admission diagnosis of TBI admitted to the ICUs of two university-affiliated trauma centres in Australia within 72 hours of injury were screened to meet the following inclusion criteria: (i) age > 18 and < 65 years; (ii) non-penetrating TBI, defined by a brain injury due to mechanical forces and the presence of haemorrhage, contusion, swelling, compression of basal cisterns or herniation on cerebral CT scan; (iii) requirement for mechanical ventilation (iv) serum alanine transferase level < 100 iU/l.

The exclusion criteria were: (i) the use of any pharmacological or physical intervention that could modify temperature within the previous 6 hours or clinician intent to modify body temperature using any pharmacological or physical intervention; (ii) body temperature < 36°C or > 38.9°C; (iii) pre-specified risk factors for paracetamol toxicity (S1 Table) (iv) TBI not expected to require intensive care management for 72 hours after randomisation; (v) GCS of 3 with fixed dilated pupils, or moribund patient expected to die within 24 hours; (vi) confirmed or suspected pregnancy.

### Treatment allocation and blinding

Randomisation was conducted using a computer-generated list of random numbers (SAS v9.2) prepared by an investigator with no clinical involvement in the patient and no access to trial data until the data analysis was complete. The study was stratified according to participating institution in a 1:1 ratio using permuted block sizes of 4, with investigators and clinicians unaware of block size.

At each study site, independent individuals who were not involved in the study or clinical activities, prepared study solutions according to randomisation codes; the study solutions were blinded, by the use of an opaque shroud to conceal differences in product packaging, although the two solutions were macroscopically indistinguishable.

Eligible patients were randomised to receive an intravenous infusion of 100mls of either 1-gram of intravenous paracetamol (Perfalgan, Bristol Myers Squib, Mulgrave, Victoria, Australia) or 0.9% sodium chloride (Pfizer, West Ryde, New South Wales, Australia) (saline) over 30 minutes, every four hours, for 72 hours.

Each dose of intravenous paracetamol solution contained mannitol (3.85g), cysteine hydrochloride, sodium phosphate dihydrate, sodium hydroxide and hydrochloric acid.

Participants, healthcare providers, data collectors, outcome adjudicators, data analysts and investigators were all unaware of treatment allocation during the conduct of the study.

### Study treatment and process measures

As 6g/day of paracetamol is higher than the recommended maximum daily dose of 4g/day daily, we monitored hepatic toxicity daily during study treatment and at 7 days after commencement of study treatment.

Study treatment would be ceased if serum alanine transferase exceeded 250 iU/L[28] or if the attending clinician suspected paracetamol toxicity, following which the intervention would be unblinded to allow consideration of the administration of intravenous n-acetyl cysteine. Additional stopping criteria included the development of clinically significant hypotension (see safety outcomes below for definitions).

Core body temperature was measured hourly using an intravesical temperature probe during the study intervention period unless specific contraindications were present, such as confirmed or suspected urethral injury or renal failure (defined as urine output < 0.3mls/kg/hr for greater than 4 hours). Alternative methods of temperature measurement included tympanic, axillary and nasopharyngeal measurements that were adjusted by -0.2, +0.6 and +0.3°C respectively, based on previous unpublished validation data by our group (S2 Table).

We collected daily information on the use of phenytoin as an indicator of associated hepatic enzyme induction that may result in increased paracetamol metabolism[29].

Pharmacological agents given with the intent of reducing body temperature, such as non-steroidal anti-inflammatory drugs, were not to be used during the study intervention period, unless directed by the treating clinician. Similarly, physical cooling interventions were not be used for a temperature of ≤ 38°C if intracranial pressure was within normal limits. Physical cooling could be considered for elevations of ICP above 20 mmHg despite standard management as directed by the treating clinician[30].

### Outcomes

The primary outcome was the absolute difference in mean core temperature during the 72-hour study treatment period.

Secondary outcomes were the difference in the mean number of hours on each day that physical cooling was used; the difference in the mean arterial pressure, and the difference in the mean intracranial pressure during the 72-hour study treatment period.

Tertiary outcomes, evaluated at day 28 after randomisation, included ICU and hospital length of stay, and hospital mortality.

Safety outcomes included the incidence of hypotension during the 72-hour study treatment period and the incidence of hepatic abnormalities during the first 7 days after randomisation. Hypotension was defined as > 1 episode of either mean arterial pressure < 50 mmHg, systolic blood pressure < 90 mmhg, or, cerebral perfusion pressure < 50 mmHg respectively, occurring within 60 minutes of study drug administration, and occurring for > 15 minutes. Hepatic abnormalities were defined as serum alanine transferase or aspartate aminotransferase values above twice the upper limit of normal; bilirubin level above the normal range or an international normalised ratio greater than or equal to 1.5.

### Sample size

The initial sample size calculation was based on a similar phase II randomised controlled trial in acute ischaemic stroke[27] that informed a predicted reduction of 0.5°C (standard deviation [SD] 0.6°C) 72 hours after commencement of intravenous paracetamol. Allowing for a 20% loss to follow-up (due to death or early discharge), a study population of 80 patients was selected at a significance level of 0.05 and power of 0.90.

We chose to investigate a change in temperature of 0.5°C[2], as intravenous paracetamol may have higher bioavailability then enteral preparations in critically ill patient populations[31]. In addition, we considered this difference to be clinically significant based on data from a cohort study of patients with acute stroke that reported a 5% to 10% absolute lower risk of poor outcome with this effect[32], and a subsequent cohort study that reported a two-fold increase in the risk of death with each 1°C elevation of temperature above 37°C[33].

### Modifications of the protocol after trial commencement

In May 2011, after 3 patients had been recruited, the inclusion criteria were modified to remove the requirement for a Glasgow Coma Score of between 3 and 8 and to extend the recruitment period from within 48 hours of injury. The modified inclusion criteria added the requirement for mechanical ventilation, and allowed for recruitment within 72 hours of injury. These changes were made to improve the generalisability of the study and to increase recruitment.

In November 2012, for recruitment and funding considerations during the conduct of our trial, we amended the analysis method to a longitudinal model to analyse all hourly temperature values during the 72-hour study treatment period. This change reduced the total sample size to between 38 and 44 patients in total, assuming a correlation coefficient of between 0.6 and 0.7 for repeated measures within an individual participant.

### Statistical methods

The statistical analysis plan was finalised prior to unblinding of the study database. Statistical analyses were conducted at the George Institute for Global Health. The principal investigator had full access to all of the data in the study and the writing committee takes responsibility for the integrity of the data and the data analysis.

Discrete variables were summarised as frequencies and percentages; percentages were calculated according to the number of patients for whom data were available, and where values were missing, the denominator was stated. Continuous variables have been summarised using standard measures of central tendency and dispersion, either mean and SD, or median and interquartile range (IQR) as appropriate.

The primary outcome, continuous secondary outcomes (mean arterial pressure and intracranial pressure) and safety outcomes (liver function abnormalities) were analysed using a repeated-measure analysis of variance with fixed effects of treatment using the baseline value as a covariate; no imputation for missing values due to death or early discharge was done. The dichotomous variable (hourly use of physical cooling) was presented as relative risk (RR) and compared between groups using a generalised linear model with a binomial distribution and logarithmic link[34]. Mortality rates at day 28 were compared using a Fisher exact test. Average ICU and hospital length of stay were compared at day 28 using a two sample Wilcoxon rank sums test. Statistical significance was evaluated by calculating 95% confidence intervals (95%CI) and a p-value <0.05 was assumed to represent statistical significance.

A p*ost hoc* analysis of the primary outcome, adjusted for pre-randomisation age, GCS and pupillary abnormalities was conducted. A sensitivity analysis of the primary outcome that excluded patients that did not have exclusively intravesical temperature measurement was also conducted.

## Results

### Study population

**Fig 1:**
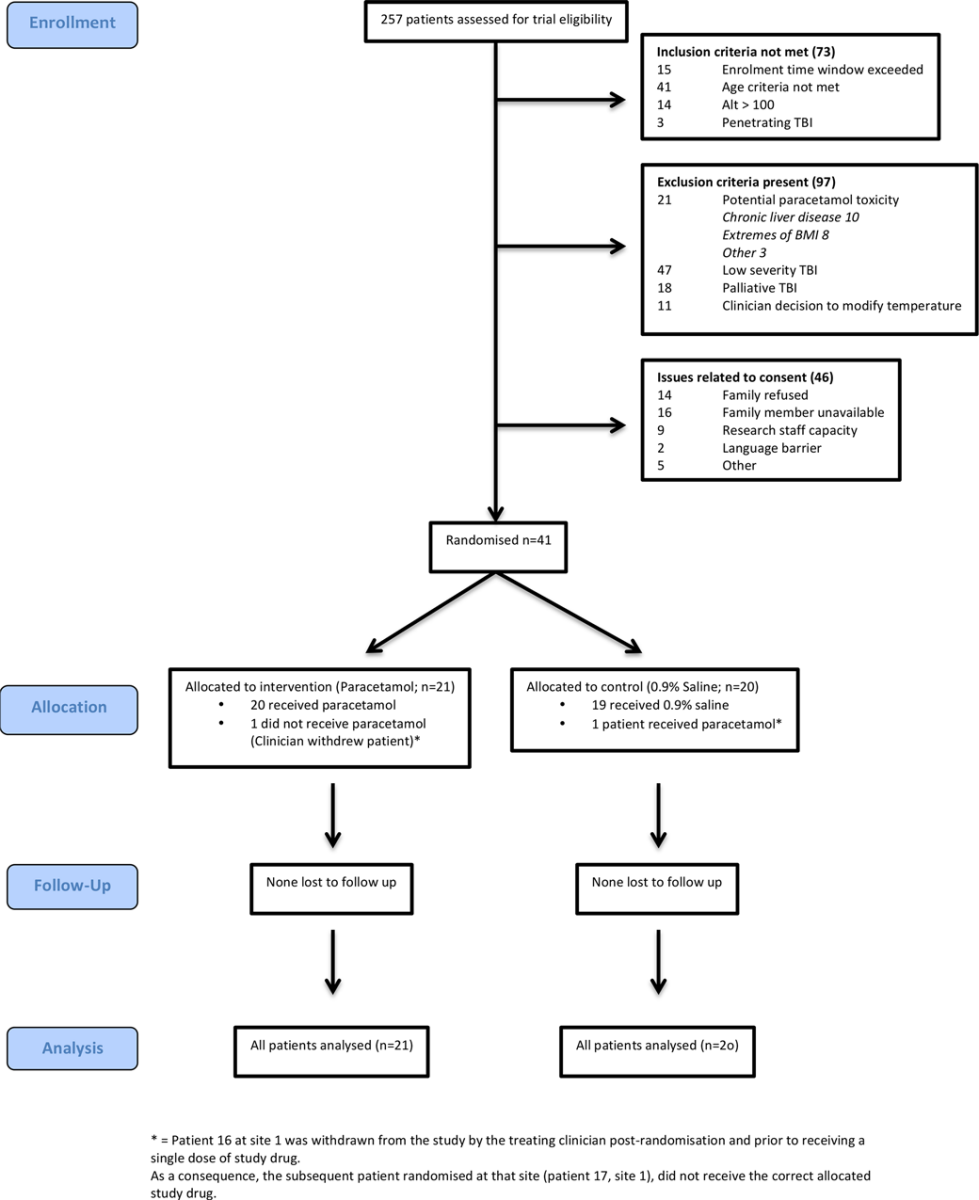
Flow of participants through the trial.

Screening and enrolment occurred between November 2010 and December 2013. Two hundred and fifty seven patients were evaluated for trial eligibility and 41 patients were randomised (Fig 1).

There was no difference in baseline characteristics between the two groups (Table 1). The mean body temperature at randomisation in the paracetamol and saline group was 37.3±0.8°C and 37.6±0.6°C respectively.

**Table 1:** Demographic characteristics of patients at and prior to randomisation.

		Paracetamol* (n = 21)	Saline* (n = 20)	Total (n = 41)
**DEMOGRAPHICS**				
Age/years: mean (SD)		33 (16)	33 (18)	33 (17)
Male Sex: n (%)		18/21 (86)	15/20 (75)	33/41 (81)
Weight/kg: mean (SD)		83 (10)	83 (18)	83 (14)
**INJURY CHARACTERISTICS**				
Type of injury: n/N (%)	Motor Vehicle	11/21 (51)	11/20 (55)	22/41 (54)
	Fall	4/21 (19)	7/20 (35)	11/41 (27)
	Other	6/21 (29)	2/20 (10)	8/41 (20)
Major extracranial injury: n/N (%)		5/19 (26)	8/18 (44)	13/37 (35)
Time from Injury to ED/hours: median (IQR)		1:47 (0.50–3.26)	2:42 (0.59–3.33)	2.09 (0.59–3.26)
Time from ED to ICU/hours: median (IQR)		5:35 (3:59–8.43)	4:25 (3:24–9.05)	5:25 (3:43–8:54)
ICU admission source n/N (%)	Emergency department	5/21 (24)	7/20 (35)	12/41 (29)
	Operating theatre	12/21 (57)	13/20 (65)	25/41 (61)
	Other	4/21 (19)	0/20 (0)	4/41 (10)
**PRE-RANDOMISATION CHARACTERISTICS**				
Time: ICU admission to randomisation/hours: median (IQR)		33:40 (22:00–42:45)	36:59 (16:45–53:44)	34:17 (19:27–52:30)
Temperature/°C:	Median (IQR)	37.6 (36.9–37.9)	37.4 (37.2–38.0)	37.5 (37.0–38.0)
	Mean (SD)	37.3 (0.8)	37.6 (0.6)	37.4 (0.7)
Mean arterial pressure/mmHg: mean (SD)		91 (12)	88 (9)	90 (10)
Vasopressor n/N (%)		5/21 (24)	6/20 (30)	11/41 (27)
Pupil responsive to light n/N (%)	Right	18/21 (86)	20/20 (100)	38/41 (93)
	Left	18/21 (86)	20/20 (100)	38/41 (93)
GCS: median (IQR)		6 (3–7)	7 (3–9)	6 (3–7)
ICU admission APACHE II score, mean (SD)		18 (4)	17 (6)	17 (5)
Alanine aminotransferase U/l: median (IQR)		37 (28–73)	34 (21–47)	36 (25–63)
Bilirubin: median (IQR)		11 (9–19)	10 (7–15)	11 (9–18)
International Normalised Ratio: median (IQR)		1.2 (1.1–1.3)	1.2 (1.1–1.2)	1.2 (1.1–1.3)
Osmotic intervention n/N (%)		10/21 (48)	7/19 (37)	17/40 (43)
Use of hyperventilation n/N (%)		5/21 (24)	1/19 (5)	6/40 (15)
Use of thiopentone n/N (%)		0 (0)	0 (0)	0 (0)
Neurosurgical Operative intervention n/N (%)		9/19 (48)	11/19 (58)	20/38 (53)
Evacuation of mass lesion (%)		7/20 (35)	9/20 (45)	16/40 (40)
Decompressive craniectomy (%)		6/20 (30)	5/20 (25)	11/40 (28)
Use of extracorporeal circuit n/N (%)		1/21 (5)	0/20 (0)	1/41 (2)
Use of steroid n/N (%)		2/21 (10)	0/20 (0)	1/41 (5)

*There were no significant differences between study groups in any of the measured baseline characteristics.

A severity of illness score using the Acute Physiology and Chronic Health Evaluation II (APACHE II[39]) was calculated with information from the first 24 hours after ICU admission. Scores on the APACHE II range from 0 to 71, with higher scores indicating more severe disease and a higher risk of death.

Abbreviations: IQR: interquartile range; SD: standard deviation; ED: emergency department; ICU: intensive care unit; GCS: Glasgow coma score; ICP: intracranial pressure.

### Process measures

The median (IQR) number of doses of study drug received by the paracetamol and control groups was 18 (17–18) and 18 (16–18) respectively (P = 0.85); 33/41 (80%) of patients received 15 or more doses of study drug: 17/21 in the paracetamol group and 16/20 in the saline group (S3 Table).

During the study intervention period, 2798 temperature measurements were made in 41 patients (median 73 [67–76] per patient). Five patients were unable to have intravesical temperature measurements for some or all of the study intervention period: of these, 2 had tympanic or axillary temperature measurements and 1 had nasopharyngeal temperature measurements.

In the paracetamol group 13/20 patients underwent intracranial pressure monitoring, compared to 15/20 patients in the control group.

Phenytoin was used in 14/21 and 19/20 patients in the paracetamol and saline groups respectively.

### Outcomes

**Fig 2:**
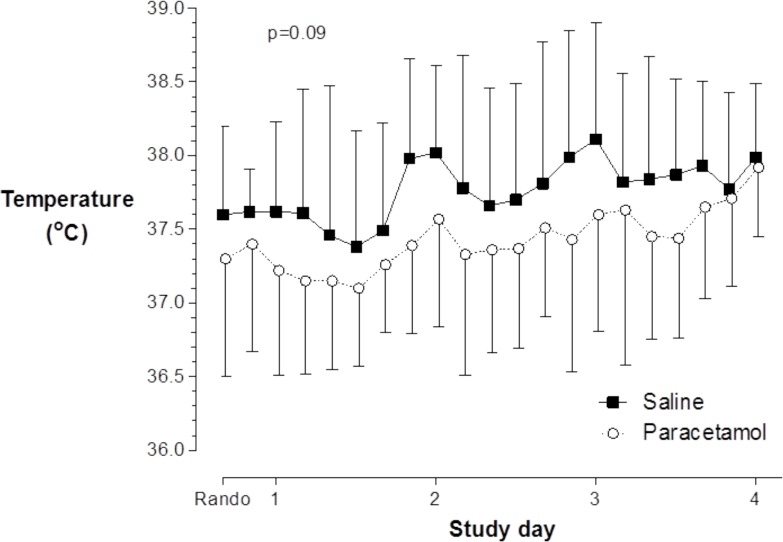
Primary outcome: mean and standard deviation (SD) temperature plotted against time during the study intervention period for paracetamol (n = 21) and saline control group (n = 2). For clarity, data are presented as mean and upper SD for saline, and mean and lower SD for paracetamol, at 4-hourly time-points following the time of randomisation (Rando), and until 4 hours after the final dose of study drug.

The mean ± SD temperature during the study period was 37.4±0.5°C and 37.7±0.4°C in the paracetamol and saline groups respectively (mean difference -0.3°C; 95%CI -0.6 to 0.0; P = 0.09) (Fig 2 and S4 Table).

The mean ± SD number of hours per day that physical cooling was used during study treatment was 2.2 ± 0.7 hours in the paracetamol group and 3.8 ± 1.2 hours in the saline group (RR 0.6; 95%CI 0.3 to 1.3; P = 0.17) (Table 2). The mean arterial pressure during study treatment was 90.4 ± 1.6 and 93.2 ± 1.6 mmHg in the paracetamol and saline groups respectively (mean difference -2.8 mmHg; 95% CI -7.3 to 1.8; P = 0.23). The mean intracranial pressure during study treatment was 12.8 ± 1.0 and 14.4 ± 0.9 mmHg in the paracetamol and 0.9% saline groups respectively (mean difference -1.5mmHg; 95% CI -4.4 to 1.3; P = 0.27).

**Table 2:** Primary and secondary outcomes.

	Paracetamol (n = 21)	Saline (n = 20)	Mean difference* or relative risk** (n = 41) (95%CI)	P-value
Mean temperature/°C (SD)	37.4 (0.5)	37.7 (0.4)	-0.3 (-0.6 to 0.0)*	0.09
(†Adjusted) Mean temperature/°C (SD):	37.5 (0.9)	37.8 (0.9)	-0.3 (-0.7 to 0.0)*	0.09
Intracranial pressure/mmHg (SD)	12.8 (1.0)	14.4 (0.9)	-1.5 (-4.4 to 1.3)*	0.2669
Mean arterial pressure/mmHg (SD)	90.4 (1.6)	93.2 (1.6)	-2.8 (-7.3 to 1.8)*	0.2260
Number of hours/day that physical cooling was used (SD)	2.2 (0.7)	3.8 (1.2)	0.6 (0.3 to 1.3)**	0.1726

† Adjusted for age, GCS and pupillary response and with baseline temperature as a covariate.

* Mean difference.

** Relative risk.

Abbreviations: CI: confidence intervals; SD: standard deviation; GCS: Glasgow coma score.

The overall day-28 mortality rate was 9.8% (4/41): 14.3% (3/21) in the paracetamol group and 5% (1/20) in the saline group (P = 0.32) (Table 3).

**Table 3:** Tertiary outcomes.

	Paracetamol (n = 21)	Saline (n = 20)	Total (n = 41)	P-value
Intensive care length of stay (days): median (IQR)	13.0 (7.0–15.0)	12.0 (6.0–15.0)	12.0 (6.0–15.0)	0.84
Hospital length of stay (days): median (IQR)	36.5 (23.0–48.0)	29.0 (20.0–41.0)	32.0 (22.5–47.0)	0.34
Hospital mortality (%)	3/ 21 (14.3)	1/ 20 (5.0)	4/ 41 (9.8)	0.61

Abbreviations: IQR: inter-quartile range.

After *post hoc* adjustment for pre-randomisation age, GCS and pupillary abnormalities, the mean temperature of the paracetamol and saline groups was 37.5°C (SD 0.9) and 37.8°C (SE 0.8) respectively (mean difference -0.3°C; 95% CI -0.7 to 0.0; P = 0.09). A sensitivity analysis excluding patients that were unable to have intravesical temperature measurement for the duration of study intervention did not alter the interpretation of the primary outcome.

### Safety

There was no episode of systemic or cerebral hypoperfusion in either group and no difference in the incidence of hepatic abnormalities (Table 4 and S5 Table) between the two study groups.

**Table 4:** Safety outcomes: incidence of abnormalities of alanine transferase, aspartate aminotransferase, bilirubin and the international normalised ratio.

	Paracetamol (n = 21)	Saline (n = 20)	Total (n = 41)	P-value
Abnormal alanine transferase (%)	8/ 21 (38.1%)	7/ 20 (35.0%)	15/ 41 (36.6%)	1.00
Abnormal aspartate aminotransferase (%)	12/ 21 (57.1%)	12/ 20 (60.0%)	24/ 41 (58.5%)	1.00
Abnormal bilirubin (%)	7/ 21 (33.3%)	4/ 20 (20.0%)	11/ 41 (26.8%)	0.48
Abnormal international normalised ratio (%)	1/ 21 (4.8%)	0/ 20 (0.0%)	1/ 41 (2.4%)	1.00

Abnormality was defined as any value > twice the upper limit of normal for ALT/AST, for bilirubin as any value above the normal range and for INR as any value ≥ 1.5.

## Discussion

### Summary of principal findings

The early, routine intravenous administration of 6 grams/day of paracetamol did not significantly reduce core temperature in patients with TBI compared to placebo.

### Strengths

We mitigated the risk of ascertainment bias through randomisation, allocation concealment, and masking of treatment assignments. The primary outcome was not subject to observer bias. We finalised the statistical analysis plan and conducted all analyses before unmasking of study groups assignments.

Although the number of patients was small, we obtained a substantive dataset of over 2700 temperature measurements that allowed a clear comparison between paracetamol and placebo, thereby providing determination of the effect size.

We used a high dose and intravenous preparation of paracetamol that may influence the generalisability of our findings, particularly in regions where intravenous paracetamol is not widely used. However, we have reported preliminary safety data for this dose of paracetamol in the TBI patient population and our results are supported by larger body of evidence in patients with stroke reporting safety of this dose via the enteral route[2, 27, 35].

Our study contributes to an increasing body of literature on temperature control using paracetamol and physical cooling during critical illness for neurological disorders[1–5] and severe infections[36].

### Limitations

Our findings are generalisable to patients with severe traumatic brain injury that require mechanical ventilation; these inclusion criteria were chosen to define a patient population that would be likely to receive a complete course of study treatment.

The use of phenytoin[29] may have reduced the efficacy of paracetamol; however this was a pragmatic study and phenytoin is used to prevent early seizures after TBI[37].

The lack of protocolisation of physical cooling in the study confounds the interpretation of the primary outcome; the administration of paracetamol was associated with a non-significant reduction in the use of physical cooling and this may have lead to an underestimation of the effect of paracetamol on temperature. Future studies in this area may need to consider this design issue. Heterogeneity in the opinions of clinicians and the absence of high quality evidence precluded a strict protocolised approach in our study protocol.

Although our study was underpowered to detect an effect size of less than 0.5°C[27], it is possible that a smaller effect may have a clinically important consequence[2, 3].

### Comparison with relevant findings from other published studies

The effect of regular paracetamol on core temperature after traumatic brain injury in our study is consistent with the effect on temperature reported in similar trials in stroke. The Acetaminophen In Stroke (PAIS) study compared the effect of 6g/day of enteral paracetamol to placebo in reducing disability and death and reported that although paracetamol reduced body temperature by 0 26°C (95% CI 0 18 to 0 31), there was no significant difference in disability or death[2]. For the subgroup of patients with a body temperature at baseline between 37 and 39°C, there was an improvement (adjusted odds ratio 1 43; 95% CI 1 02 to 1 97), compared to patients with a baseline temperature of 36–37°C. The PAIS-2 study was designed to further examine this exploratory finding and recently closed recruitment in October 2014[3].

### Clinical and research implications

The burden of severe TBI is increasing in low- and middle-income countries and is associated with increased urbanisation and mechanisation but decreasing in high-income countries due to preventative public health measures[38]. Pyrexia is common after TBI[6–10] and there is a biological rationale that reducing pyrexia after TBI may have the potential to improve patient-centred outcomes[2, 8, 10–16]. Clinical trials are needed to evaluate if interventions given to avoid pyrexia reduce disability and death after TBI[17–21].

## Conclusion

The early administration of 6g/day of intravenous paracetamol did not result in a significant reduction in core body temperature in patients with TBI and was not associated with clinically important adverse effects.

## Supporting Information

S1 Checklist (DOC)

S1 Protocol (DOC)

S1 Table Exclusion criteria related to risk of paracetamol toxicity. (DOCX)

S2 Table Unpublished validation study of 46 critically ill patients, evaluating the relationship between tympanic, axillary and nasopharyngeal temperature measurement, and intravesical temperature measurement. (DOCX)

S3 Table Study Drug administration. (DOCX)

S4 Table Mean core temperature at 24, 48 and 72 hours after randomisation. (DOCX)

S5 Table Safety outcomes. (DOCX)
