# Supplementary material for: The Effect of Paracetamol on Core Body Temperature in Acute Traumatic Brain Injury: A Randomised, Controlled Clinical Trial
Source: PLoS One. 2015 Dec 17;10(12):e0144740. doi: 10.1371/journal.pone.0144740 (PMC4683067; doi:10.1371/journal.pone.0144740)
Supplement: S1 Protocol — (DOC) [file pone.0144740.s002.doc]

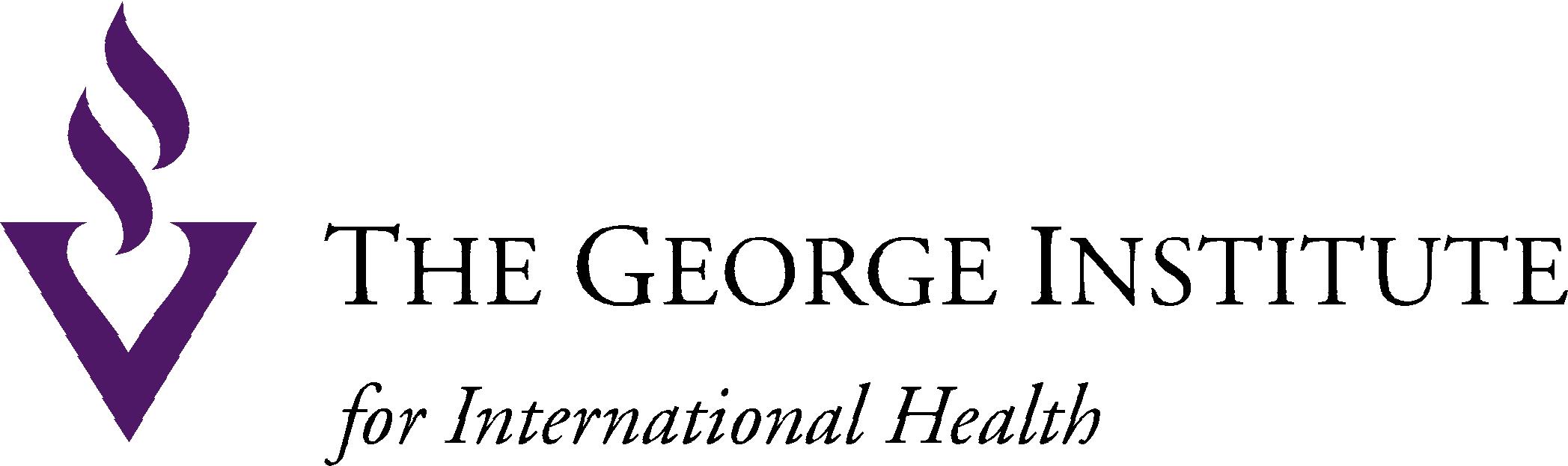

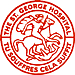

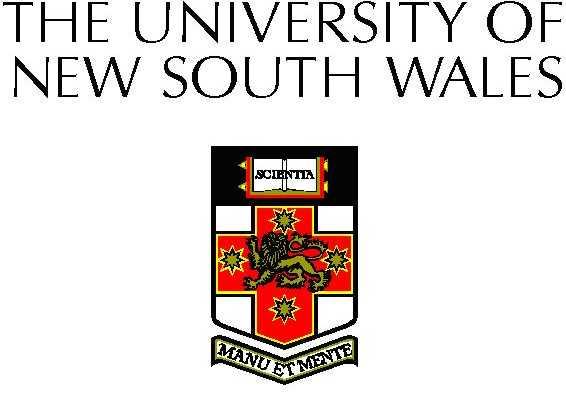


**
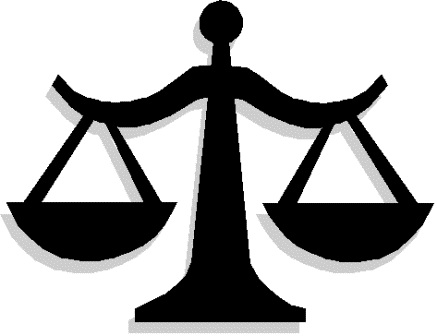
**

**The Paracetamol AfteR Traumatic brain InjurY study**

**The PARITY study**

A multi-centre phase 2b randomised controlled trial investigating the efficacy and safety of intravenous paracetamol in reducing core body temperature after traumatic brain injury.

**CLINICAL TRIAL PROTOCOL**

**PROTOCOL NAME: PARITY**

**PROTOCOL VERSION: 004**

**PROTOCOL DATE: 17/05/10**

- ANZCTRN: ACTRN12609000444280
- NSW Ethical Approval SWAHS HREC, Nepean Campus: Study 09/20 – HREC 09/NEPEAN/43: 14/10/2009
- NSW Guardianship Tribunal Approval (14/2009): 13/04/10
- Supported by a Project Grant (09/008) from the Australia and New Zealand College of Anaesthetists: November 2008

**TABLE OF CONTENTS**

1. GENERAL INFORMATION 6

1.1 Title 6

1.2 Chief investigator 6

1.3 Coordinating and data management centre 6

2. BACKGROUND INFORMATION 7

2.1 Background 7

2.2 The clinical uncertainty of temperature management after TBI 7

2.3.1 Phase 2 and phase 3 studies of paracetamol 8

2.3.2 Australia and New Zealand Consensus Guidelines on 9

Paracetamol toxicity - “supra-therapeutic” paracetamol ingestion

2.4 Rationale for the PARITY study 10

2.4 Trial feasibility 10

3. TRIAL AIM & HYPOTHESIS 12

3.1 Primary aim 12

3.2 Secondary aims 12

3.3 Tertiary aims 12

4. TRIAL DESIGN 12

5. ASSESSMENT OF EFFICACY 13

5.1 Specification of the efficacy parameters 13

5.1.1 Primary outcome 13

5.1.2 Secondary outcomes 13

5.1.3 Tertiary outcomes 13

5.1.4 Parameters of efficacy and safety 13

6. SELECTION OF PARTICIPANTS

6.1 Inclusion criteria 14

6.2 Exclusion criteria 14

7. RANDOMISATION AND ALLOCATION OF TREATMENT 14

8. TREATMENT OF PARTICIPANTS 15

8.1 Study treatment 15

8.2 Dosage regimen 16

8.3 Concomitant treatment during trial period 16

8.4 Withdrawal of study treatment 16

8.4.1 Withdrawal of study treatment criteria 17

8.4.2 Management for patients for whom study treatment has been 17

withdrawn

8.5 Unblinding 17

9. DURATION OF PARTICIPATION 18

10. SUPPLIES AND ACCOUNTABILITY PROCEDURES 18

10.1 Study treatment inspection and accountability 18

10.2 Disposal of used and unused study treatment 18

11. STUDY TERMINATION 19

12. STUDY PROCEDURES 19

12.1 Data collection 19

12.2 Screening Log 19

12.3 Data management of patients for whom study treatment has been 20 withdrawn

12.4 Method of data collection 20

12.5 Case Report Forms 20

13. ASSESSMENT OF SAFETY 20

13.1 Adverse Event and Serious Adverse Event Reporting 20

13.1.1 Definitions 20

13.1.1.1 Adverse events (AEs) 21

13.1.1.2 Serious adverse events (SAEs) 21

13.1.1.3 Suspected unexpected serious adverse reaction (SUSARs) 21

13.2 Study safety reporting 21

13.2.1 Notification of immediately reportable adverse events (SUSARSs) 21

13.2.2 Notification of immediately reportable AEs 22

13.2.2.1 Notification of treatment related AEs 22

14. Independent Data Monitoring Committee 23

15. STATISTICS 23

15.1 Sample size and justification 23

15.1.1 Sample size 23

15.1.2 Justification 23

15.2 Statistical methods 23

15.3 Pre-identified subgroup analyses 23

15.4 Interim analyses 24

15.5 Criteria for the termination of the trial 24

16. DIRECT ACCESS TO DATA & DOCUMENTS 24

17. QUALITY CONTROL AND QUALITY ASSURANCE MONITORING 24

17.1 Responsibilities of the investigator 24

17.2 Responsibilities of the co-coordinating centre 25

17.3 Source document requirements 26

17.4 Management of protocol deviations 26

18. ETHICAL CONSIDERATIONS 26

18.1 Ethical Principles 26

18.2 Independent Ethics Committee 26

18.3 Informed Consent 27

18.3.1 Process of obtaining consent where the patients are highly 28

dependent on medical care

18.3.1.1 Informed consent from participant 28

18.3.1.2 Informed consent from substitute decision maker 28

19. DATA HANDLING AND RECORD KEEPING 28

20. FINANCING AND INSURANCE 29

20.1 Funding 29

20.2 Participating Centres agreement 29

21. PUBLICATION POLICY 29

22. PROJECT TIMELINE 30

23. REFERENCES 31

24. APPENDICES 35

# LIST OF DEFINITIONS AND ABBREVIATIONS

Interventions that may effect core temperature:

- Pharmacological therapies include paracetamol and NSAIDs
- Physical therapies include bedside fans, ice packs, cold intravenous fluids, cooling blankets, cooling helmets/wraps and intravenous cooling catheters.

AE Adverse Event

AIS Acute Ischaemic Stroke

ANZCA Australia and New Zealand College of Anaesthetists

ANZICS-CTG Australian and New Zealand Intensive Care Society- Clinical Trials Group

ALT Alaninine Transferase

CPP Cerebral Perfusion Pressure

CI Confidence interval

CRF Case report form

CTN Clinical Trial Notification

ICH International Conference on Harmonisation of Technical Requirements for Registration of Pharmaceuticals for Human Use

ICU Intensive Care Unit

MAP Mean Arterial Pressure

NEAF National Ethics Application Form

PAIS Paracetamol for Acute Ischaemic Stroke study

SAE Serious Adverse Event

SDM Substitute Decision Maker

SUSAR Suspected Unexpected Serious Adverse Reaction

TBI Traumatic Brain Injury

TGA Therapeutic Goods Administration

# 1. GENERAL INFORMATION

This protocol has been developed according to guidelines from The Australian Clinical Trial Handbook (1).

## Title

The Paracetamol AfteR Traumatic brain InjurY study (The PARITY study)

## Chief investigator

Name: Dr Manoj Saxena

Title: Staff Specialist in Intensive Care,

St. George Hospital,

Address: Gray Street, Kogarah, NSW 2217

Contact Number: (02) 9113 3373

Fax number: (02) 9113 3971

Email: msaxena@george.org.au

## Coordinating and data management centre

Name: Department of Intensive Care Medicine

Address: St. George Hospital,

Gray Street,

Kogarah, NSW 2217,

Australia

Research Methods Centre:

The George Institute for International Health

Division of Critical Care,

Level 7, 341 George Street,

Sydney, NSW 2001,

Australia

### 2. BACKGROUND INFORMATION

## 2.1 Background

Worldwide, traumatic brain injury (TBI) is the leading cause of disability in adults under 40 years of age and 10 million people each year are hospitalized with TBI (2). The Australasian Traumatic Brain Injury Study (ATBIS), a prospective, cohort study of 636 patients with TBI requiring intensive care admission, demonstrated that at 12 months the mortality rate was 26.9% and an unfavorable outcome was present in 41.2 % of patients (dichotomized Glasgow outcome score) (3). Similar rates of morbidity and mortality were reported in a recent large international study of over 2500 patients (4). The Global Burden of Disease Project (2) predicts a substantial increase (50-60%) in disability and death following road traffic accidents (a major cause of TBI globally), with the incidence increasing in low- and middle-income countries and falling in high-income countries. However, although, the long-term disability in survivors has major social and economic implications for society, there remains a significant lack of coherent evidence about effective therapies in the acute care of patients with TBI. Therapies that are widely available and inexpensive warrant investigation, as they may be applicable to patients in low- and middle-, as well as high-income countries, if efficacy and safety were to be demonstrated.

In acute ischaemic stroke (AIS), observational studies have demonstrated that raised body temperature (>37.5 C) in the first few days after stroke is associated with larger infarct volume, poorer functional outcome and increased mortality (5-7). Further analysis of the Copenhagen stroke study suggests that a 1 C increase in body temperature is associated with a 2.2 increase in mortality (6). An additional meta-analysis of observational data also suggested that a raised body temperature after stroke or TBI was associated with adverse morbidity and mortality (8). These data from stroke and TBI suggest that interventions that induce small temperature differences after neuronal injury warrant further investigation in randomised-controlled trials.

**2.2 The clinical uncertainty of temperature management after TBI**

Certain animal data (9, 10) have suggested that induced hypothermia may reduce neuronal damage following brain injury. Observational clinical studies also suggest an association between a raised body temperature after TBI and adverse morbidity and mortality (8, 11, and 12). There is evidence to support the use of induced hypothermia following cardiac arrest in adults (body temperature reduction to 32 to 34 C) and perinatal birth asphyxia (body temperature reduction to 35 C) in neonates (13, 14). Pathophysiologically, TBI differs from cardiac arrest and neonatal birth asphyxia in that the primary insult is direct neuronal trauma, often complicated by associated ischaemic/hypoxic secondary insults. The positive results of temperature reduction after cardiac arrest and neonatal birth asphyxia have not been replicated in patients with TBI, despite a number of interventional trials. A Cochrane review (15) concluded that there is no evidence that hypothermia (defined as interventions that reduce core body temperature to at least 34 to 35ºC) may be beneficial in the treatment of TBI. In addition, this review suggested that there is a statistically significant increase in pulmonary infections with the intervention of hypothermia compared to controls.

Induced, systemic hypothermia remains a complex, unproven intervention that can only be applied to a small proportion of the more severely injured TBI patients where advanced pre-hospital trauma care, intensive care facilities and skilled staff are readily available. Conversely, less-intensive interventions maintaining normothermia may, be applicable to a much larger proportion of patients with TBI, particularly in low- and middle-income countries. (16). Some of these interventions, such as surface cooling or pharmacological therapies are commonly used in the absence of any evidence or recommended treatment guidelines. A recent systematic review concluded that there was no evidence that normothermia after TBI improves patient outcomes (17).

There is therefore no Grade A or B evidence (large or small randomized, controlled clinical trials) that supports the practice of targeting normothermia after TBI. However, despite this scientific uncertainty, analysis of the control arms of the hypothermia trials (15) and several recent surveys (18-20) suggest that normothermia has become the default therapy, with the occasional use of systemic hypothermia. The Brain Trauma Foundation (21) in addition does not make any recommendation regarding normothermia after TBI further suggesting an absence of evidence to guide clinical practice. Therefore there exists clinical uncertainty about the effect of interventions that target normothermia on patient-centred outcomes after TBI and research is warranted.

**2.3.1 Phase 1, Phase 2 and Phase 3 studies with paracetamol**

Paracetamol is a potent inhibitor of prostaglandin synthesis within the central nervous system and this presumably accounts for its antipyretic and analgesic properties. The peak plasma concentration of intravenous paracetamol (compared to oral paracetamol) is greater (30mg/ml vs. 24 mg/ml) and occurs earlier (15 minutes vs. 60 minutes) (22, 23). However after the first hour, and, up to 24 hours, the plasma concentrations remained similar. Problems with administration and absorption of oral paracetamol in head injured patients may preclude the reliable attainment of adequate serum levels of the drug by this route. Bioavailability of rectal suppositories is approximately 80% of that of the tablets, with a slower rate of absorption and maximal plasma concentrations achieved about 2-3 hours after administration. There is also considerable variation in the peak plasma concentrations following rectal administration (24) and there is likely to be variability in rectal absorption in head injured patients as there may be concomitant abdominal injuries, relative gut hypoperfusion and contraindications to the rectal route. Hence although the intravenous route adds to the cost of the intervention ($3.30 vs. $0.02 for 1 gram of paracetamol), the intravenous route is preferred for this study, despite the slight increase in cost.

Paracetamol is mainly conjugated in the liver and then excreted in the urine. The plasma half-life is 1.5-3 hours. A single dose of 140mg/kg or more has the potential to saturate the glucuronide conjugation pathway and hepatic toxicity may result. In the phase 2 and the phase 3 study, 6 g/day was not associated with significant hepatic morbidity (25-27).

Animal studies suggest that doses of 100-150mg/kg are safe (28, 29), but both intra- (30) and inter-species (31) differences limit the external validity of these results to patient populations. A number of healthy volunteer studies have demonstrated the safety of single doses of 15-90 mg/kg (32, 33, 34-37) and two additional studies have demonstrated the safety of both 6 grams and 8 grams per day for 3 days in human volunteers (38, 39). Two retrospective studies (singles dose of 15mg/kg and total dose of 4 grams per day) (40, 41) and one prospective case series (42) have reported on the use of IV paracetamol for fever control in severe TBI. There are no randomised, controlled clinical trials evaluating the safety or efficacy of intravenous paracetamol in TBI.

Two previous phase 2 and one phase 3 randomised placebo-controlled studies in AIS have demonstrated that 6g of paracetamol given daily safely reduces body temperature by 0.26°C (95% CI: 0.18-0.31) compared to placebo (25-27). The same studies failed to show a significant difference in temperature between a lower dose of paracetamol (500mg, 6 hourly) and placebo and also ibuprofen and placebo. These studies suggest that only the higher dose of paracetamol is efficacious in reducing body temperature after AIS (25).

**2.3.2 Australia and New Zealand Consensus Guidelines on paracetamol toxicity - “supra-therapeutic” paracetamol ingestion**

It is important to differentiate between intentional paracetamol overdose (single dose or repeated doses over a few hours – total dose exceeding either 10 grams or 140mg/kg) and paracetamol taken in divided doses for pain relief but exceeding the recommended 4g/24 hours (supra-therapeutic ingestion or accidental overdose). It is acknowledged in the recent Consensus Statement (“Guidelines for management of paracetamol toxicity in New Zealand and Australia”) that there is “little evidence to guide risk assessment” for supra-therapeutic ingestion (43). The Consensus Statement suggests hepatic injury may be associated with the use of paracetamol for the following doses:

- 200mg/kg or 10g (whichever is less) over a 24 hour period
- > 150mg/kg or 6g (whichever is less) for the preceding 48 hour period
- > 100mg/day or 4g (whichever is less) in patients with predisposing risk factors (chronic ethanol misuse, use of enzyme-inducing drugs, prolonged fasting, dehydration)

The Consensus Guidelines suggest a treatment algorithm based on measurement of serum paracetamol levels (> 10mg/L) and serum ALT levels (if ALT level exceeds normal values) at the time of presentation.

Evaluation of the two studies (44, 45) that are referenced in the Consensus Guidelines to support the treatment algorithm for “supra-therapeutic” paracetamol ingestion would lead to a Grade E recommendation (Non randomized case series or expert opinion) according to the criteria developed for grading evidence by Sackett et al (46). The first study was an American prospective cohort study (Level 4 evidence – Non randomized, historical controls, expert opinion) (44). The second study was a randomized, single-blind, placebo-controlled study of 145 healthy adults in 2 American inpatient clinical pharmacology units, but the treatment dose of paracetamol evaluated was a daily dose of 4 grams per 24-hours (45). Although this study is potentially graded at Level 2 evidence (small randomized, controlled clinical trial; moderate to high risk of false positive/negative results), the study investigates the effects of 4 grams of paracetamol per 24 hours and does not give information pertaining to the dose that we plan to investigate (6 grams per 24 hour period). The authors of the Consensus Guidelines in summarizing these two studies acknowledge that there is “little evidence to guide risk assessment”, and, that “the threshold for the reported dose that causes toxicity has been made deliberately and conservatively low”.

The Consensus Guidelines do not include the data available from the two Phase 2 (total 150 patients) and one phase 3 (1400 patients) randomized, controlled, blind clinical trials in AIS (25-27). These sources of evidence would be graded as level 2 (phase 2 studies) and level 1 (phase 3 study) and together would constitute a Grade B recommendation (one large and 2 small randomized, controlled clinical trials) that the use of 6 grams per day is safe. These three studies have established that a dose of 6 grams of paracetamol a day for 3 days is effective in reducing core temperature after AIS and has a similar safety profile to placebo on liver function tests, liver failure and sepsis. The same data demonstrate that a smaller daily dose does not reduce core temperature (25).

Therefore, having evaluated the recommendations of the Consensus Guidelines, we would argue that there exists an ethical and scientific imperative to provide high-grade evidence to guide practice in this area of clinical uncertainty. The only way to evaluate the safety and efficacy of 6 grams of paracetamol per day for 3 days in patients with traumatic brain injury is within well-designed, rigorously conducted randomised, controlled clinical trials.

**2.4 Rationale for the PARITY study**

There are several arguments against the routine or *ad hoc* prescription of pharmacological interventions that may induce normothermia for patients with TBI. First there is a concern because treatment with these agents may mask infection and lead to delayed detection of sepsis. Secondly, side-effects may occur such as hypotension or liver toxicity (paracetamol). Further as demonstrated in the Cochrane systematic review it has not been established that active reduction of body temperature after TBI results in improved patient outcome (15, 17), and, additionally, it is not known whether paracetamol does in fact have a clinically meaningful effect on body temperature after TBI.

Therefore there is clinical uncertainty regarding the true effect of targeting normothermia with paracetamol after TBI and it is possible that these interventions may have a clinical benefit, may have no effect, or, may indeed have a harmful effect on patients. The only way to rigorously evaluate these interventions is by performing well designed randomised, placebo controlled clinical studies.

Our study is therefore designed to answer the question of the efficacy of 6 g of intravenous paracetamol given for 3 days after TBI in reducing body temperature whilst evaluating safety.

**2.5 Trial feasibility**

The investigators have an established track record of excellence in the conduct of both small- and large-scale randomised-controlled trials in intensive care in Australia and New Zealand. The Saline and Albumin Fluid Evaluation study (47) and the Normoglycaemia in Intensive Care study (48), conducted by the Australian and New Zealand Intensive Care Society Clinical Trials Group (ANZICS CTG) and the George Institute, are regarded as a benchmark for trials in Critical Care Medicine. Together these trials randomised 13,000 patients. Additionally, both principal centres collaborated in the CAT study (a phase 2 randomised controlled trial of noradrenaline vs. adrenaline in the reversal of shock in critically ill patients) (49) and all centres involved have a highly developed infrastructure that can support and accommodate the proposed study.

- The trial has a simple, pragmatic, high-quality methodological design with allocation concealment, blinding, minimisation of ascertainment bias and loss to follow-up, analysed on an intention-to-treat basis.
- The centres involved admit in excess of 350 patients per year with TBI and have the ability to randomise the 80 patients required to ensure adequate statistical power.

**3 TRIAL AIM & HYPOTHESIS**

## 3.1 Primary aim

The primary aim of the PARITY study is to determine whether the early administration of intravenous paracetamol reduces core body temperature following severe traumatic brain injury compared to placebo (normal saline). The null hypothesis assumes no difference in core body temperature after the administration of intravenous paracetamol compared to placebo following severe TBI.

The study will establish whether a definitive phase III trial is feasible and justifiable, and will provide essential information on the cost and likely sample size for such a trial.

## 3.2 Secondary aims

The secondary aims of the PARITY study are:

1. To determine if there is any difference in the use of physical therapies in patients randomised to intravenous paracetamol compared to normal saline placebo.
2. To determine if there is a difference in the 3-day time-weighted mean temperature or the 3-day temperature curves in patients randomised to intravenous paracetamol compared to normal saline placebo.
3. To determine if there is any difference in the mean intracranial pressure and mean arterial pressure on day 1, day 2 and day 3 in patients randomised to intravenous paracetamol compared to normal saline placebo.
4. To determine if there is any difference in the incidence of hypotension (systolic blood pressure < 90 mmhg or Mean arterial pressure < 50 mmhg for > 15 minutes) in patients randomised to intravenous paracetamol compared to normal saline placebo (21).
5. To determine if there is any difference in the incidence of cerebral hypoperfusion (cerebral perfusion pressure < 50 mmhg for > 15 minutes) (21).
6. To determine if there is any difference in the incidence of abnormalities of conjugated, synthetic or ischaemic liver function (peak values of alanine transferase, the international normalised ratio and aspartate aminotransferase) in patients randomised to intravenous paracetamol compared to normal saline placebo.

## 3.2 Tertiary aims

1. To determine the pharmacokinetic profile of intravenous paracetamol
   1. Mean peak paracetamol concentration after a single dose: samples at baseline, 30 minutes, 45 minutes, 90 minutes and 240 minutes
   2. Mean paracetamol concentration 240 minutes after the final dose.
2. To determine if there is any difference in intensive care unit length of stay, hospital length of stay or mortality at 28 days in patients randomised to intravenous paracetamol compared to normal saline placebo.

**4 TRIAL DESIGN**

The PARITY study will be a prospective, phase 2b, multi-centre, blinded, randomised placebo-controlled trial, designed to answer the research question of the efficacy (and safety) of intravenous paracetamol in reducing body temperature after TBI.

# 5 ASSESSMENT OF EFFICACY

## 5.1 Specification of the efficacy parameters

### 5.1.1 Primary outcome

The primary outcome measure for the study will be core body temperature measured at 72 hours.

### 5.1.2 Secondary outcomes

- Use of physical therapies
- Core and tympanic temperature
- Intracranial pressure
- Systolic blood pressure
- Mean arterial pressure
- Incidence of abnormalities of conjugated, ischaemic and synthetic liver function (peak values of alanine transferase, aspartate aminotransferase and the international normalised ratio)

### 5.1.3 Tertiary outcomes

- Serum paracetamol levels
- ICU, hospital length of stay and mortality

### 5.1.4 Parameters of efficacy and safety

- Use of physical therapies
- Core temperature measurement after final dose of study drug at 72-hours
- Core and tympanic temperature measurements during the administration of study drug (hourly)
- Intracranial pressure measurement (6 hourly during period of study drug administration) – only in patients that require intracranial pressure monitoring as part of their usual care.
- Systolic blood pressure (6 hourly, and, at time of samples for paracetamol levels)
- Mean arterial pressure (6 hourly, and, at time of samples of paracetamol levels)
- Conjugated, ischaemic and synthetic liver function tests (baseline, daily during study drug administration and until day 7 after randomisation)
- Incidence of hypotension (within 60 minutes of study drug administration) – SBP < 90 mmhg or MAP < 50 mmhg for > 15 minutes (21)
- Incidence of cerebral hypoperfusion (within 60 minutes of study drug administration) - cerebral perfusion pressure < 50 mmhg for > 15 minutes (21)
- Serum paracetamol levels (and blood pressure) at baseline and 30, 45, 90 and 240 minutes after a single dose of intravenous study drug
- Serum paracetamol levels (and blood pressure) 240 minutes after final dose of intravenous study drug.
- Length of stay in ICU (Day 28 Summary)
- Length of stay in hospital (Day 28 Summary)
- Mortality and cause of death (Day 28 Summary)

# 6 SELECTION OF PARTICIPANTS

## 6.1 Inclusion criteria

Patients treated in the intensive care are eligible for inclusion in the study if **ALL** of the following criteria are met:

- Written informed consent has been obtained from the patient’s next of kin
- Age > 18 and < 65
- Non penetrating head injury with a post-resuscitation GCS between 3 and 8 and an abnormal CT head (defined by the presence of haemorrhage, contusion, swelling, compression of basal cisterns or herniation)
- Within 48 hours of injury
- Presence (or imminent placement) of arterial cannula
- Alanine transferase level < 100 (44, 45)

## 6.2 Exclusion criteria

Patients are excluded from the study if **ONE OR MORE** of the following criteria are met:

- Suspected paracetamol overdose or allergy to paracetamol
- Confirmed or suspected pregnancy
- Use of pharmacological or physical intervention that reduces body temperature in the 6-hour period prior to randomisation.
- Clinician decision to institute any pharmacological or physical intervention that modifies body temperature
- Body temperature at time of recruitment less that 36°C or greater than 38.9°C
- History of chronic liver disease or chronic alcohol abuse
- Suspected malnutrition: BMI < 18 kg/m2 or weight < 60 kg
- BMI > 35 kg/m2
- Renal failure with serum creatinine > 200
- Haemodynamic instability defined as systolic blood pressure < 90 mmhg or requirement for noradrenaline or adrenaline exceeding 20mcg/minute
- Use of hepatic enzyme inducers, except for phenytoin
- Minor head injury: either normal CT head or not expected to be in intensive care for 72 hours
- GCS = 3 with fixed dilated pupils
- Moribund patient expected to die within 24 hours

**7 RANDOMISATION AND ALLOCATION OF TREATMENT**

Preparation of the study treatment (shrouding of study treatment) will be carried out by an independent person at each study site.

Randomisation will be achieved using computer generated random numbers. Randomisation will be stratified according to participating institution.

All staff and patients will be blinded as to the treatment allocation.

# 8 TREATMENT OF PARTICIPANTS

## 8.1 Study treatment

Eligible patients will be randomised to receive either:

- 1 gram of intravenous paracetamol
- 0.9% sodium chloride (saline).

The study treatments are macroscopically identical and will be supplied in an identical format. The formulation of intravenous paracetamol contains other ingredients (mannitol, cysteine hydrochloride, sodium phosphate dihydrate, sodium hydroxide and hydrochloric acid) to buffer the solution to a neutral pH. 3.85 g of mannitol is included, but this is a small amount compared to the dose of 1g/kg of mannitol that has been investigated in acute head injury. Therefore to reduce the cost and complexity of the placebo we propose to use normal saline for the placebo arm.

All patients in this study will have an arterial line, which will facilitate serum paracetamol samples to be taken without any discomfort to the patient. A serum paracetamol level will be checked prior to study treatment and each patient will subsequently require 5 additional samples of 5 millilitres of blood to be taken for the analysis of serum paracetamol levels.

Temperature measurements will be by a core temperature probe. Temperature will be measured prior to administration of study drug, hourly during the period of administration of study drug and until 4 hours after the last dose of study medication.

All clinician’s involved in the prescription of blinded study treatment must read the Investigators Brochure/Product Information (Appendix B) which provides detailed information about the composition, indications, side effects, suggested dosage and contraindications of the study treatments. Study drug must be stored below 30 degrees Celsius and not refrigerated.

**8.2 Dosage regimen**

The product information (Appendix B) states that intravenous paracetamol should be given over 15 minutes. However, we propose that the study medication should be administered over 30 minutes. This is an additional safety feature to reduce the incidence of presumed paracetamol induced hypotension that has been reported (incidence between 1/1000 and 1/10,000) with the administration of intravenous paracetamol. The dose will be repeated every 4 hours for a total of 18 doses following randomisation.

Weight must be recorded at randomisation. The patient’s weight can be obtained either by direct measurement or estimated. The patient’s weight and method of estimation will be recorded.

## 8.3 Concomitant treatment during trial period

Concomitant pharmacological and physical treatments that affect body temperature are to be *PREFERENTIALLY AVOIDED* during the study intervention period, but discretion rests with the treating clinician after consideration of sections 8.3.1, 8.3.2, 8.3.2.1 and 8.3.2.2:

**8.3.1 Concomitant pharmacological therapies**

Concomitant pharmacological therapies such as paracetamol and NSAIDs may be used if the treating clinician feels that pharmacological therapies are clinically indicated. In this instance the study medication must be stopped (section 8.4.1) and treatment may then commence with the pharmacological therapy.

**8.3.2 Concomitant Physical therapies**

Concomitant physical therapies may be used if the treating clinician feels that they are clinically indicated. If physical therapies are used then study medication should continue.

**8.3.2.1 Isolated core temperature above 38°C**

Physical therapies should ideally not be used for an isolated elevated temperature (intracranial pressure within normal limits) unless temperature exceeds 38°C and the aim of treatment with the physical therapy is to reduce core temperature to below 38°C. However, preference resides with the treating clinician.

**8.3.2.2 Intracranial pressure > 20 mmhg**

If ICP remains above 20 mmhg for greater than 15 minutes as measured by a calibrated probe with an optimal trace, despite ‘standard management’, then physical therapies may be used by the treating clinician to reduce temperature (‘standard management’ may include 30° bed head elevation, optimal positioning of neck collars/tapes, PaO2 > 80 mmhg, PaCO2 35-40 mmhg, optimal sedation/analgaesia, CSF drainage and removal of mass lesions).

Apart from temperature management and measurement (8.1), patient management will be otherwise unaffected, and the treating clinicians will be free to provide whatever other medical care is deemed necessary for the patient.

## 8.4 Withdrawal of study treatment

The patient (or the Person Responsible for the patient) may withdraw from study treatment at any time, irrespective of the reason. The treating clinician may also withdraw the patient from study treatment if it is felt that withdrawal from study treatment is in the patient’s best interests. The reason for withdrawal of treatment will be recorded in the CRF. All treatment discontinuation should also be recorded by the investigator in the source notes.

### 8.4.1 Withdrawal of study treatment criteria

Study treatment must be stopped under the following conditions:

1. The treating clinician feels that pharmacological therapy to reduce body temperature is clinically indicated (section 8.3).
2. Alanine transferase > 250 (44, 45).
3. > 1 episodes of hypotension or cerebral hypoperfusion (defined as MAP < 50 mmhg, systolic blood pressure < 90 mmhg, and, CPP < 50 mmhg respectively) occurring within 60 minutes of study drug administration, and, occurring for > 15 minutes (21).

If (2) occurs and is thought to be related to the study drug, then the participant *may* be unblinded (see 8.6) and treatment *may* commence with n-acetyl cysteine as per the Consensus Guidelines for paracetamol toxicity (43):

1. Initial infusion: An initial dose of 150 mg/kg* of N-acetylcysteine diluted in 200 mls of 5% glucose and infused over 15 to 60 minutes
2. Second infusion: Initial infusion is followed by a continuous infusion of 50 mg/kg* of N-acetylcysteine in 500 mls of 5% glucose over the next 4 hours
3. Third infusion: Second infusion is followed by a continuous infusion of 100 mg/kg* of N-acetylcysteine in 1000 mls of 5% glucose over the next 16 hours

* = based on lean bodyweight

### 8.4.2 Management of patients for whom study treatment has been withdrawn

Patients withdrawn from the randomised treatment for any reason will be followed up according to the study follow up schedule and analysed according to the intention-to-treat-principle unless the patient or their legal surrogate has specifically withdrawn consent to follow up data being used.

A withdrawal form will be completed if a patient or their legal surrogate withdraws consent for data collection.

## 8.5 Unblinding

Unblinding of participants should only be performed when knowledge of the treatment allocation will influence the participant’s management in a significant fashion.

The precise reason for unblinding must always be provided, together with details of the name of the clinician making the decision, the date and time the decision was made and any supporting documentation that supports the decision (such as laboratory reports).

In any case of unblinding, the follow-up schedule of data collection should be maintained to enable full analysis of all patient data on an intention-to-treat basis.

The investigator will contact the coordinating centre if they consider there is a need for unblinding and this will be adjudicated by the Study Management Committee (Appendix C)

**9 DURATION OF PARTICIPATION**

The maximum duration of study participation is 4 weeks.

All patients will participate in the active treatment phase and will be followed until the earliest of any of the following:

- Completion of the follow-up period at 4 weeks post-randomisation
- Withdrawal of consent, by subject or legal surrogate
- Patient death

# 10 SUPPLIES AND ACCOUNTABILITY PROCEDURES

## 10.1 Study treatment inspection and accountability

The investigator or delegate at each hospital will be responsible for receiving, inspecting, and documenting the study drugs prior to placement in the ICU store. The investigator or delegate will carry out an inventory and acknowledge receipt of all shipments of the study treatments. Documentation of study drug distribution, receipt, use and disposal will be kept to enable comprehensive tracking and reconciliation of all study treatments, used or unused.

Study treatments must be kept in a locked area with restricted access. The study treatments must be stored and handled in accordance with the manufacturer’s instructions. The investigator or delegate will also keep accurate records of the quantities of the investigational products dispensed, used, and returned by each participant.

## 10.2 Disposal of used and unused study treatment

All used partially used and unused study drugs should be returned to the original container once the infusion or the need for infusion has finished.

Once a patient has left the ICU or died, the contents of all containers used by that patient should be returned to the research co-ordinator. The research co-ordinator may at this stage complete the data entry for this patient for this ICU episode and cross check the study drug that is identified on the hospital charts against the study drug that has been used from the study bags.

The containers must then be sealed and stored until the study monitor conducts a monitoring visit to the ICU. At this monitoring visit, a check of the study drugs that has been recorded on the database and notes made on the patient log will be checked against the study drug in the leftover bottles.

The study monitor will periodically check the supplies of study treatments held by the investigator to verify accountability of all study treatments used. At the end of the monitoring visit, the research co-ordinator will transfer all the checked containers to the agreed area where the bags will be disposed of according to local guidelines.

At the conclusion of the study, all unused study treatments will be destroyed unless other arrangements have been approved by the co-coordinating centre. The co-coordinating centre will verify that a final report of study treatment accountability is prepared and maintained in the investigator study file.

# 11 STUDY TERMINATION

The study may be terminated at any time at the request of the study management committee, the Investigator, or a regulatory authority, with proper and timely notification of all parties concerned. The Independent Ethics Committee will be informed promptly and the co-ordinating centre or the investigator will supply reason(s) for the termination or suspension, as specified by the applicable regulatory requirements. Otherwise, the study is considered terminated upon completion of all patient treatments and evaluations.

# 12 STUDY PROCEDURES

## 12.1 Data collection

Data collection will be by paper case report forms (CRF) at each site. Each site will forward the original CRF to the coordinating centre, keeping a copy of the CRF at the site. The coordinating centre will take responsibility for the data management of the study. This includes programming and data management support of the database during the study.

The table below provides a summary and time schedule of the data to be collected in the trial CRF.

| **Form No.** | **Period of study** | **Data collection** |
| --- | --- | --- |
|  | Randomisation | Patient identifiers, eligibility criteria |
| **1** | Baseline Information | Source and date of admission to ICU, ICU admission diagnosis, age, APACHE II score, post resuscitation GCS, presence or absence of ICP monitoring. |
| **2** | Days 1 - 7 | Temperature, intracranial pressure, synthetic and conjugated liver function, serum paracetamol levels, episodes of hypotension |
| **3** | Day 28 | Length of stay (intensive care, hospital) and mortality |

## 12.2 Screening Log

The screening log is designed to monitor patient recruitment at each of the study centres. A screening log of all patients evaluated for enrolment in the study will be compiled monthly by research co-coordinators at each study site. The log will record all patients screened, either randomised into the study or considered ineligible for the study. Additionally, the reason patients were excluded or the reasons eligible patients were not enrolled will be recorded in the log. A copy of the log should be retained in the investigator’s study files. The co-coordinating centre will compile a cumulative screening log monthly, using information from each study site.

## 12.3 Data management of patients for whom study treatment has been withdrawn

In patients in whom study treatment has been discontinued, the follow-up schedule must continue unchanged for all randomised participants unless the patient or their representative has specifically withdrawn consent to follow up.

Efforts will be made to follow all study participants for 4 weeks, irrespective of their adherence to the randomized therapy.  All losses to follow-up with reason will be reported to the coordinating centre and reviewed by the Study Management Committee.

Patients withdrawn from randomised study treatment will be included in the final analysis on an intention to treat basis.

## 12.4 Method of data collection

Data collected while the patient is in the ICU will be from the patient medical record by the research co-ordinator. Follow-up that occurs outside of the ICU will be conducted by the research co-ordinator within the hospital (if the participant is still in hospital). Research co-ordinators conducting follow-up will be blinded to the treatment allocation at all times.

## 12.5 Case Report Forms

The case report form (CRF) will be developed by the coordinating centre and made available to the participating sites as paper CRFs. Research co-coordinators will enter all required data described in the protocol onto the CRFs directly from the source data.

Information recorded in the CRF should accurately reflect the subject’s medical/hospital notes. Information must be completed in the case report form as soon as it is made available for recording. The intent of this process is to improve the quality of the clinical study by providing prompt feedback to the investigators on the progress of the data submitted and to enhance the ability to collect early safety information in a more timely fashion to fully comply with the intent of GCP requirements.

# 13 ASSESSMENT OF SAFETY

## 13.1 Adverse Event and Serious Adverse Event Reporting

### 13.1.1 Definitions

#### **13.1.1.1 Adverse events (AEs)**

Adverse events are defined as any untoward medical occurrence in a patient administered an investigational intervention and which does not necessarily have to have a causal relationship with this treatment (50)

#### **13.1.1.2 Serious adverse events (SAEs)**

Serious adverse events are defined as any untoward medical occurrence that meets one of more of the following criteria:

- Results in death
- Is life-threatening
- Requires inpatient hospitalisation or prolongation of existing hospitalisation
- Results in persistent or significant disability/incapacity

The classification of ‘serious adverse event’ is not related to the assessment of the severity of the adverse event. An event that is mild in severity may be classified as a serious adverse event based on the above criteria.

If there is any doubt whether an event constitutes an SAE, this event should be reported as an SAE.

#### **13.1.1.3 Suspected unexpected serious adverse reaction (SUSARs)**

SUSARs are defined as a serious adverse event for which there is some degree of probability that the event is an adverse reaction to the administered drug, and the adverse reaction is unexpected.

## 13.2 Study safety reporting

It is recognised that the patient population in the ICU will experience a number of aberrations in laboratory values, signs and symptoms due to the severity of the underlying disease and the impact of standard therapies. These will not necessarily constitute an adverse event or serious adverse event unless they require significant intervention or are considered to be of concern in the investigator’s clinical judgement.

Hence in this study reporting of adverse events will be restricted to:

- Serious adverse events that are unexpected and considered to be study treatment related (SUSARs);
- Other AEs thought to be related to treatment and not already collected as part of the routine data collection;

### 13.2.1 Notification of immediately reportable adverse events (SUSARs)

Only SAEs that are unexpected and thought to be related to treatment (SUSARs) are subject to expedited reporting and should be reported to the co-ordinating centre within 24 hours of study staff becoming aware of the event. Minimum information to report will include:

- Patient initials and study number
- Nature of the event
- Commencement and cessation of the event
- An investigator’s opinion of the relationship between study involvement and the event (unrelated, possibly, probably or definitely related).
- Whether treatment was required for the event and what treatment was administered.

SUSARs must be reported on the relevant adverse event form and must also be discussed with the co-ordinating centre staff or chief investigator. The telephone is the preferred method as it assures both parties that the message has been received and will be acted on. Contact details will be provided to all study centres for this purpose. The co-ordinating centre staff or Chief Investigator will review these events.

The co-ordinating centre staff will be responsible for following-up SUSARs to ensure all details are available. The co-ordinating centre is also responsible for alerting other participating centres of the report of a SUSAR and reporting SUSARs to the regulatory authorities (Therapeutic Goods Administration in Australia within 7 days with an additional report within 8 days).

It is the responsibility of each site investigator at each site to inform their local Independent Ethics Committee of all such events which occur at their site, in accordance with local requirements. Copies of any reporting and correspondence to and from the local Independent Ethics Committee should also be sent to the co-ordinating centre.

For a SUSAR the Principal Investigator or treating clinician must make a decision about the need to unblind the study fluid for that patient. Wherever possible, it is preferable to maintain the blind. However, if knowledge of the study treatment type is required to treat the adverse event quickly and correctly, then unblinding may be required. The Therapeutic Goods Administration advises that retention of blinding is considered “understandable when the fatal or serious outcome is identical or closely resembles the primary efficacy endpoint of the study” (51). These outcomes would be considered to be ‘disease-related’ and exempted from expedited reporting”.

### 13.2.2 Notification of immediately reportable adverse events

Other reportable adverse events are those adverse events that are thought to be related to treatment but not already collected as part of the routine data collection.

#### **13.2.2.1 Notification of treatment related AEs**

Treatment-related adverse events not already collected in the routine data collection should be reported on the relevant form and submitted to the co-ordinating centre as soon as possible. These will be reviewed by the co-ordinating centre staff or Chief Investigator and recorded in a safety database, which will be monitored by co-ordinating staff on a regular basis.

Investigators are advised to refer to the Investigator’s Brochure for information on adverse events and adverse reactions associated with the study treatments.

**14 INDEPENDENT DATA MONITORING COMMITTEE**

A Data Monitoring Committee (DMC), independent from the investigators, will perform an ongoing review of predefined safety parameters and overall study conduct. The DMC will be comprised of experts in clinical trials and methodology. The DMC’s primary function is to ensure the safety and welfare of the patients. The DMC will review data on patient characteristics, specified outcomes and reportable adverse events at predetermined intervals during the study or as deemed appropriate by the DMC

# 15 STATISTICS

## 15.1 Sample size and justification

### 15.1.1 Sample size

The planned sample size is 80 patients.

### 15.1.2 Justification

The sample size for this study has been calculated taking into account the following assumptions:

To be able to detect a difference in body temperature at 72 hours of 0.5°C, with a significance level of 0.05 and power of 0.90, assuming a standard deviation of 0.6°C, we will need 31 patients in each treatment group. The clinical trials of the efficacy of paracetamol in acute ischaemic stroke used a similar sample size calculation (10, 11). Our study is a similar small phase 2b study that focuses on efficacy and safety and we acknowledge the need to balance sample size with budget. Allowing for some variability in the figures for the sample size calculation we believe that a total study population of 80 patients would allow us to answer the question of whether intravenous paracetamol is a safe intervention after TBI and if it is efficacious in reducing core body temperature.

## 15.2 Statistical methods

A detailed statistical analysis plan will be developed before completion of recruitment and published in a public domain before commencement of analysis:

The study results will be analysed on an intention-to-treat basis. The primary outcome data will be presented as an absolute mean difference in temperature between the intervention and control groups. The secondary outcome of intracranial pressure will also be presented as a comparison between the two groups of mean values. We will compare the 28 day mortality in the 2 groups using a log rank test. All tests of significance will be at the 5% level and 2-sided. The statistical analysis will be carried out at the George Institute for International Health.

## 15.3 Pre-identified subgroup analyses

Pre-planned sub-group analyses are planned relating to temperature at baseline > 37°C and the use of phenytoin.

## 15.4 Interim analyses

The Data Monitoring Committee will perform at least one interim analysis when 50% of participants (40 patients) have reached day 28 follow-up.

Additional reviews of the data may be performed at the discretion of the Data Monitoring Committee.

The Data Monitoring Committee will also review summaries of all serious adverse events and deaths that occur during the study.

## 15.5 Criteria for the termination of the trial

The DMC will independently assess the safety and efficacy data and determine appropriate stopping criteria.

# 16 DIRECT ACCESS TO DATA & DOCUMENTS

The study may be audited by government regulatory authorities, local Independent Ethics Committees or qualified representatives of St. George Hospital, or, the George Institute for International health as permitted by the regulation (51). Therefore access to medical records, other source documents such as ICU charts and other study related files must be made available at all study sites for monitoring and audit purposes during the course of the study and after its completion.

Participants will not be identified by name, and confidentiality of information in medical records will be preserved. The confidentiality of the participant will be maintained unless disclosure is required by regulations.

# 17 QUALITY CONTROL AND QUALITY ASSURANCE MONITORING

## 17.1 Responsibilities of the investigator

The investigator agrees to perform the clinical trial in accordance with this clinical trial protocol, International Conference on Harmonisation of Technical Requirements for Registration of Pharmaceuticals for Human Use (ICH) and the applicable regulatory requirements (52). The investigator is required to ensure compliance with all procedures required by the clinical trial protocol and with all study procedures provided by the co-coordinating centre.

The investigator agrees to provide reliable data and all information requested by the clinical trial protocol in an accurate and legible manner according to the instructions provided. The investigator agrees to allow representatives of the co-coordinating centre to have direct access to source documents.

## 17.2 Responsibilities of the co-coordinating centre

The co-coordinating centre is responsible for taking all reasonable steps to ensure the proper conduct of the clinical trial protocol.

Prior to initiation of the study at each participating site, the co-ordinating centre will be responsible for providing adequate training to the Principal Investigator and study personnel. The training will cover all aspects of the study protocol and procedures and will include practical training in completing the CRF and the study materials. All study materials will be provided at or before the training sessions.

The Chief Investigator (CI) will monitor this study. During the trial, the sites will be contacted, through monitoring visits, letters or telephone calls, by the CI to review study progress, investigator and patient compliance with study protocol requirements and any emergent problems. The main duty of the study CI is to help the investigator and the coordinating centre maintain a high level of ethical, scientific, technical and regulatory quality in all aspects of the trial.

Site monitoring visits will be performed periodically and in accordance with the Monitoring Plan. The investigator and study personnel will assist the CI by providing all appropriate documentation, and being available to discuss the study. These monitoring visits will include but not be limited to review of the following aspects:

- Adherence to the protocol including consistency with inclusion and exclusion criteria;
- The completeness and accuracy of the case record forms (CRFs) and source documentation, patient informed consent;
- Patient recruitment and follow-up;
- Adverse Event documentation and reporting;
- Study treatment allocation;
- Patient compliance with the study treatment regimen;
- Study treatment accountability;
- Compliance with regulations.

At completion of the trial, a final monitoring and close out visit will be conducted by the CI in accordance with the Monitoring Plan. Secure facilities for the storage of study data for 15 years will also be re-checked at this visit.

## 17.3 Source document requirements

According to the ICH guidelines for Good Clinical Practice (52), the monitoring team must check the Case Report Form entries against the source documents. The purpose of source documents is to document the existence of the participant and substantiate the integrity of the study data collected. Source documents include the original documents related to the trial, to medical treatment, and to the history of the subject. Adequate and accurate source documents allow the investigator and the site monitor to verify the reliability and authenticity of data recorded on the CRFs and ultimately to validate that the clinical study was carried out in accordance with the protocol.

## 17.4 Management of protocol deviations

A protocol deviation is an unanticipated or unintentional departure from the expected conduct of an approved study that is not consistent with the current research protocol or consent document. A protocol deviation may be an omission, addition or change in any procedure described in the protocol.

The investigator should not implement any deviation from or changes of the protocol without agreement by the study management committee and documented approval from the Independent Ethics Committee of the amendment, except where necessary to eliminate an immediate hazard(s) to trial participants. In the event of an emergency intended to eliminate an apparent immediate hazard to participants the Investigator may implement any medical procedure deemed appropriate.

Deviations from the protocol must be documented and promptly reported to the study management committee and the Independent Ethics Committee (if applicable). The report should summarize the event and action taken.

# 18 ETHICAL CONSIDERATIONS

## 18.1 Ethical Principles

This trial will be conducted in accordance with the principles laid down by the ICH guidelines for Good Clinical Practice (52) and the applicable local regulatory requirements.

## 18.2 Independent Ethics Committee

The Principal Investigator is responsible for submitting this protocol to the Independent Ethics Committee. In Australia, an initial application requesting approval to conduct this study will be submitted using a National Ethics Application Form (NEAF) to a lead Human Research Ethics Committee (HREC) in NSW. In addition, a review by the Guardianship Tribunal of New South Wales may be conducted as appropriate.

Thereafter, further applications will be submitted to other Independent Ethics Committees in Australia and/or to the Hospital Research Ethics Committee at each of the participating hospitals. Each application will be submitted according to the requirements of each hospital committee, all of which are formed and are conducted in accordance with the guidelines laid down by the National and Medical Research Council of Australia (53).

The content and format of the patient and Substitute Decision Maker (SDM) information statements and consent forms will be approved by each Independent Ethics Committee and produced in line with their own guidelines and requirements. Each participating hospital will therefore use their own consent documents as approved by their local Independent Ethics Committee.

During the trial, any amendment or modification to the study protocol should be notified to the Independent Ethics Committee by the Principal Investigator and approved by the Independent Ethics Committee before implementation, unless the change is necessary to eliminate an immediate hazard to the patients, in which case the Independent Ethics Committee should be informed as soon as possible.

Each Principal Investigator will be responsible for informing the Independent Ethics Committee of any event likely to affect the safety of patients or the continued conduct of the clinical trial, in particular any change in safety.

The Principal Investigator will produce progress reports, adverse event reports, and any other required documentation to the local Independent Ethics Committee in accordance with their guidelines. Any amendments or additions to the study protocol and material must be notified to the Independent Ethics Committee by the Principal Investigator and approved by the Independent Ethics Committee.

It is the responsibility of the Principal Investigator at each participating hospital to keep an up to date record of all correspondence and applicable documentation with the local Independent Ethics Committee.

A clean copy of the consent forms and information statements that are to be used at each hospital, together with a copy of all signed consent forms and any other consent related correspondence must also be kept in a separate file for this study, in case of any future requirement for audit purposes.

## 18.3 Informed Consent

The NHMRC National Statement on the Ethical Conduct in Human Research (53) acknowledges in Chapter 4.4 that research involving patients who are heavily dependent on medical care, such as the patients in this study, is necessary to assess and improve the efficacy and safety of interventions used in their treatment.

Obtaining written and informed consent from patients who are highly dependent on medical care such as patients in ICU is difficult because Intensive Care patients are often unconscious, sedated, intubated and too ill to understand information relating to clinical trial participation.

For patients that are highly dependent on medical care, such as patients in ICU, the preparation of consent documentation and associated consent procedure must adhere to the relevant local laws and guidelines. For the PARITY study, the procedure for obtaining consent must be written and approved by the ethics committee prior to use.

The standard for obtaining consent for participation in the PARITY Study would ideally be in the form of written informed consent from the conscious and comprehending patient, prior to enrolment into the study. However, it is expected that all of the patients eligible for this study will be too unwell or sedated as part of their treatment to be considered competent to give informed consent to participate in this study.

A number of approaches to obtaining consent in this study have been developed from the guidelines in Chapter 4.4 of the National Statement (53) and also from the ANZICS Clinical Trials Group Ethics Handbook for Researchers (54) The National Statement provides guidance for such patients in sections 4.4.9 through to 4.4.14.

In accordance with the National Statement, whenever possible, consent to participating in the study should be sought from or on behalf of the patients before project related activities are undertaken.

### 18.3.1 Process of obtaining consent where the patients are highly dependent on medical care

#### **18.3.1.1 Informed consent from participant**

Where it is possible for a conscious and comprehending patient to give informed consent to take part in this study before project related activities are undertaken, the study will be explained verbally to that patient by the investigator or research coordinator and the patient will be given the opportunity to read the participant information sheet. After they have had their questions answered and if they are willing to take part in the study, they will be asked to sign the consent form. Intensive care physicians are highly experienced at caring for critically ill patients and also evaluating the competence of their patients to understand their illness and consent for therapeutic interventions. Where appropriate, a treating clinician who is not part of the study team will assess the competence of a potential participant to consent for research. If the patient is deemed competent and consents to participate, they will be given a copy of the signed and dated consent form as well as the participant information sheet and any other documentation discussed through the consent process.

#### **18.3.1.2 Informed consent from substitute decision maker**

####

If a potential participant lacks the capacity to give consent then consent should be obtained from the participant’s guardian, or person or organisation authorised by law before project related activities are undertaken (4.4.10). The criterion of who may give consent for a patient to take part in medical research (and the term with which they may be described) varies between the States and Territories of Australia. For the purposes of this protocol, the descriptor “substitute decision maker” (SDM) describes the person who is legally allowed to give consent for the patient. The procedure for obtaining consent from the SDM must be approved by the local Independent Ethics Committee prior to use.

If the SDM cannot attend the hospital to participate in the consent process within the time constraints of the study, consent for patient participation in the study may *not* be obtained over the telephone.

# 19 DATA HANDLING AND RECORD KEEPING

Folders will be provided for the research co-ordinator to file any paper documents used for any form of data collection for each patient and to store the signed and dated consent forms. A comprehensive guide to the data collection with definitions and rationale will be provided together with a paper version of the data collection forms. Paper documents will be stored in secure locked cabinets with access limited to authorized persons. All of the documents will be available in PDF format for printing. The aim is to assist the research co-ordinator to ensure high-quality data collection and data entry.

Data management will be provided by the CI. The principle means of data collection and data processing will be via original paper CRFs that will be submitted to the CI. All forms will be signed and dated by the authorised study staff and all changes made following data submission will be recorded.

When archiving or processing data pertaining to the investigator and/or to the patients, the co-coordinating centre shall take all appropriate measures to safeguard and prevent access to this data by any unauthorized third party.

The investigator must maintain confidential all study documentation, and take measures to prevent accidental or premature destruction of these documents. It is recommended that the Investigator retain the study documents at least fifteen years after the completion or discontinuation of the study. The investigator must notify the Study Management Committee prior to destroying any study essential documents following the study completion or discontinuation. If the investigator's personal situation is such that archiving can no longer be ensured by him/her, the investigator shall inform the Study Management Committee and the relevant records shall be transferred to a mutually agreed upon designee.

If any investigator retires, relocates, or otherwise withdraws from conducting the study, the responsibility for maintaining records may be transferred to the CI. All associated documentation must also be updated.

# 20 FINANCING AND INSURANCE

## 20.1 Funding

The study is an investigator-initiated study and funding has been obtained from the Australian and New Zealand College of Anaesthetists (ANZCA Project Grant 2008)

## 20.2 Participating Centres agreement

Participating Centre agreements will be signed between the participating sites and St. George Hospital and cover:

- Trial work and duration
- Obligations of the Principal Investigator
- Confidentiality
- Intellectual property
- Indemnity

As this is an investigator-initiated study carried out by staff specialists employed in the public health system, indemnity is provided by each hospital for their own investigators.

# 21 PUBLICATION POLICY

The study will be conducted in the name of the PARITY investigators. The central project coordination and data management will be by the CI at St. George.

The principal publication from the study will be in the name of the PARITY Investigators with full credit assigned to all collaborating investigators, research coordinators and institutions. Where an individuals’ name is required for publication it will be that of the writing committee, with the chair of the writing committee listed first and subsequent authors listed alphabetically.

**22 PROJECT TIMELINE**

| **Date** | **Project Milestone** |
| --- | --- |
| May-June 2009 | Draft protocol finalized |
| May-June 2009 | Finalisation of CRF and Patient information and consent document. |
| June 2009 | NEAF application to lead HREC |
| September 2009 | Site selection, initiation and induction |
| July 2010 | Commencement of recruitment |
| November 2011 | Database lock, data analysis and initial results |
| February 2013 | Publication #1 |

# 23 REFERENCES

1. Australian Government Department of Health and Ageing and Therapeutic Goods Administration. The Australian Clinical Trials Handbook. 2006; [www.tga.gov.au/ct/cthandbook.pdf](http://www.tga.gov.au/ct/cthandbook.pdf).
2. Mathers CD, Lopez AD, Murray CJL. The burden of disease by condition: data, methods and results from 2001. (Chapter 3). *The Burden of Disease and Risk Factors. New York: Oxford university Press, 2006*
3. Myburgh JA, Cooper DJ, Finfer SR, Venkatesh B, Jones D, Higgins A et al. Epidemiology and 12-month outcome after traumatic brain injury in Australia and New Zealand (2008) J Trauma;64: 854-862
4. Hukkelhoven CW, Steyerberg EW, Rampen AJ, Farace E, Habbema JD et al. Patient’s age and outcome following severe traumatic brain injury: an analysis of 5600 patients. J Neurosurg 2003;99:666-73
5. Azzimondi G, Bassein L, Nonino F, Fiorani L, Vignatelli L, Re G et al. Fever in acute stroke worsens prognosis. A prospective study. Stroke 1995;26:2040-43
6. Reith J, Jorgensen HS, Pedersen PM, Nakayama H, Raaschou HO, Jeppesen LL et al. (1996) Body temperature in acute stroke: relation to stroke severity, mortality and outcome. Lancet;347:422-5
7. Jorgensen HS, Reith J, Nakayama H, Kammersgaard LP, Houth JG, et al Potentially reversible factors during the very acute phase of stroke and their impact on the prognosis: is there a large therapeutic potential to be explored? Cerebrovasc Dis 2001;11:207-11
8. Greer DM, Funk SE, Reaven NL, Ouzounelli M, Uman GC. Impact of fever on outcome in patients with stroke and neurologic injury: a comprehensive meta-analysis. Stroke 2008;39:3029-3035
9. Busto R, Dietrich WD, Globus MYD, Ginsberg MD. The importance of brain temperature in cerebral ischemic injury. Stroke 1989;20(8):1113-4
10. Gunn AJ, Gunn TR, de Haan HH, Williams CE, Gluckman PD. Dramatic neuronal rescue with prolonged selective head cooling after ischemia in fetal lambs. Journal of Clin Investigation 1997;99(2):248-56
11. Stocchetti N, Rossi S, Zanier ER, Colombo A, Beretta L, Citerio G. Pyrexia in head-injured patients admitted to intensive care. Intensive Care Med. 2002;28:1555–1562.
12. Geffroy A, Bronchard R, Merckx P, Seince P, Faillot T, Albaladejo P et al. Severe traumatic head injury in adults: Which patients are at risk of early hyperthermia? Intensive Care Med 2004;30:785–790
13. Bernard SA, Gray TW, Buist MD, Jones BM, Silvester W, Gutteridge G et al. Treatment of comatose survivors of out of hospital cardiac arrest with induced hypothermia. NEJM 2002;346:557-63
14. Gluckman PD, Wyatt JS, Azzopardi D, Ballard R, David Edwards A, Ferriero M et al. Selective head cooling with mild systemic hypothermia after neonatal encephalopathy: multicentre, randomised trial. Lancet (2005);365:663-70
15. Sydenham E, Roberts I, Alderson P. Hypothermia for traumatic head injury. Cochrane Database of Systematic Reviews 2009, Issue 1. Art. No.: CD001048. DOI: 10.1002/14651858.CD001048.pub3.
16. Andrews PJD, Anderson EL, Saxena MK. 2007 Cooling therapies after neuronal injury – systemic hypothermia and direct brain cooling. Yearbook of Intensive Care and Emergency Medicine (Published: Springer)
17. Saxena M, Andrews PJD, Cheng A. Modest cooling therapies (35-37.5 C) for traumatic brain injury. Cochrane Database of Systematic Reviews 2007, Issue 4. Art. No.: CD006811. DOI: 10.1002/14651858.CD006811.
18. Boerrigter MG, Andrews PJD, Rhodes A, Sterz F, Friberg H, Soreide E et al. Survey of hypothermia in European intensive care units. (abstract, personal communication)
19. Jacka M, Zygun D (on behalf of the Canadian Neuro Critical Care Network) “survey of management of severe head injury in Candada” Can J Neurol Sci 2007;34(3): 307-12
20. Johnstone NJ, King AT, Protheroe R, Childs C. Body temperature management after severe traumatic brain injury: Methods and protocols used in the United Kingdom and Ireland 2006 Resuscitation;70: 254-63
21. Guidelines for the management of severe traumatic brain injury. Journal of Neurotrauma 2007;24 s1-117
22. Clissold SP. Paracetamol and phenacetin. 1986 Drugs 32(Supp 4) 46-59
23. Bristol Myers Squibb Pharmaceuticals. Perfalgan Approved Product Information. 2004
24. Rostami-Hodjegan A, Shiran MR, Ayesh R et al. A new rapidly absorbed paracetamol tablet containing sodium bicarbonate. A four way crossover study. Drug Development and Industrial Pharmacy 2002;28:523-531
25. Dippel DWJ, van Breda EJ, van Gemmert HMA, van der Worp HB, Meijer RJ, Kapelle LJ et al. Effect pf paracetamol on body temperature in acute ischemic stroke: a double blind, randomised phase 2 clinical trial. Stroke 2001;32:1607-1612
26. Dippel DWJ, van Breda EJ, van der Worp HB, , van Gemmert HMA, Meijer RJ, Kapelle LJ et al. Effect of paracetamol and ibuprofen on body temperature in acute ischemic stroke (PISA), a phase 2 double-blind, randomised, placebo-controlled trial [ISRCTN98608690] BMC cardiovascular disorders 2003;3:2
27. Den Hertog HM, van der Worp HB, Maarten H, van Gemert A, Algra A, Kappelle LA on behalf of the PAIS investigators: The Paracetamol (Acetaminophen) In Stroke (PAIS) trial: a multicentre, randomised, placebo-controlled, phase III trial: Lancet Neurology (2009);8:434-40
28. Savides MC, Oehme FW, Nash SL, Leipold HW. The toxicity and biotransformation of single doses of acetaminophen in dogs and cats. Toxicology & Applied Pharmacology. 1984;74(1):26-34
29. Hjelle JJ, Klaassen CD. Glucuronidation and biliary excretion of acetaminophen in rats. Journal of Pharmacology & Experimental Therapeutics. 1984;228(2):407-13
30. Placke ME, Ginsberg GL, Wyand DS, Cohen SD. Ultrastructural changes during acute acetaminophen-induced hepatotoxicity in the mouse: a time and dose study. Toxicologic Pathology 1987;15(4):431-8
31. Jemnitz K, Veres Z, Monostory K, Kobori L, Vereczkey L. Interspecies differences in acetaminophen sensitivity of human, rat, and mouse primary hepatocytes. Toxicology in Vitro 2008;22(4):961-7
32. Zacharias M, De Silva RK, Hickling J, Medlicott NJ, Reith DM. Comparative safety and efficacy of two high dose regimens of oral paracetamol in healthy adults undergoing third molar surgery under local anaesthesia. Anaesthesia & Intensive Care 2007;35(4):544-9
33. Silvanto M, Munsterhjelm E, Savolainen S, Tiainen P, Niemi T, Ylikorkala O et al. Effect of 3 g of intravenous paracetamol on post-operative analgesia, platelet function and liver enzymes in patients undergoing tonsillectomy under local anaesthesia. Acta Anaesthesiologica Scandinavica 2007;51(9):1147-54,
34. Gregoire N, Hovsepian L, Gualano V, Evene E, Dufour G, Gendron A. Safety and pharmacokinetics of paracetamol following intravenous administration of 5 g during the first 24 h with a 2-g starting dose. Clinical Pharmacology & Therapeutics 2007;81(3):401-5
35. Juhl GI, Norholt SE, Tonnesen E, Hiesse-Provost O, Jensen TS. Analgesic efficacy and safety of intravenous paracetamol (acetaminophen) administered as a 2 g starting dose following third molar surgery. European Journal of Pain Ejp 2006;10(4):371-7,
36. Sumida SM, Sato RL, Wong JJ, Yamamoto LG. Acetaminophen levels 4 and 7 hours after 2000 and 3000 mg single doses in healthy adults. Hawaii Medical Journal 2003;62(1):6-9
37. Treluyer JM, Tonnelier S, d'Athis P, Leclerc B, Jolivet-Landreau I, Pons G. Antipyretic efficacy of an initial 30-mg/kg loading dose of acetaminophen versus a 15-mg/kg maintenance dose. Pediatrics 2001; 108(4):E73
38. Gelotte CK, Auiler JF, Lynch JM, Temple AR, Slattery JT. Disposition of acetaminophen at 4, 6, and 8 g/day for 3 days in healthy young adults. Clinical Pharmacology & Therapeutics 2007;81(6):840-8
39. Temple AR, Lynch JM, Vena J, Auiler JF, Gelotte CK. Aminotransferase activities in healthy subjects receiving three-day dosing of 4, 6, or 8 grams per day of acetaminophen. Clinical Toxicology: The Official Journal of the American Academy of Clinical Toxicology & European Association of Poisons Centres & Clinical Toxicologists 2007;45(1):36-44
40. Brown JM, Udomphorn Y, Suz P, Vavilala MS. Antipyretic treatment of noninfectious fever in children with severe traumatic brain injury. Childs Nervous System 2008;24(4):477-83,
41. Geffroy A, Bronchard R, Merckx P, Seince P, Faillot T, Albaladejo P et al. Severe traumatic head injury in adults: Which patients are at risk of early hyperthermia? Intensive Care Med 2004;30:785–790
42. Hinz J, Rosmus M, Popov A, Moerer O, Frerichs I, Quintel M. Effectiveness of an intravascular cooling method compared with a conventional cooling technique in neurologic patients. J Neurosurg Anaesthesiol 2007;19:130-5
43. Daly FFS, Fountain JS, Murray L, Graudins A, Buckley NA. Guidelines for the management of paracetamol poisoning in Australia and New Zealand — explanation and elaboration. MJA 2008;188:296–301
44. Daly FFS, O’Malley GF, Heard K, Bogdan GM, Dart RC. Prospective evaluation of repeated supratherapeutic acetaminophen (paracetamol) Ingestion. Ann Emerg Med 2004;44:393-398
45. Watkins PB, Kaplowitz N, Slattery JT, Colonese CR, Colucci SV, Stewart PW et al. Aminotransferase elevations in healthy adults receiving 4 grams of acetaminophan daily. JAMA 2006;296:87-93
46. Sackett DL. Rules of evidence and clinical recommendations on the use of antithrombotic agents. Chest 1989;95;2S-4S
47. The SAFE study investigators. A comparison of saline and albumin for fluid resuscitation in the intensive care unit (2004) N Engl J Med ;350: 2247-56
48. The NICE-SUGAR investigators. The Normoglycemia in Intensive Care Evaluation (NICE) (ISRCTN04968275) and Survival Using Glucose Algorithm Regulation (SUGAR) Study: Intensive versus Conventional Glucose Control in Critically Ill Patients. N Engl J Med 2009;360:1283-1297
49. Myburgh JA, Higgins A, Jovanovska A, Lipman J, Ramakrishnan N, Santamaria J. A comparison of epinephrine and norepinephrine in critically ill patients. Intensive Care Medicine 2008;34:2226-34
50. Australian Government Department of Health and Ageing and Therapeutic Goods Administration. Note for guidance on clincial safety data management: Definitions and standards for expedited reporting. 2000; [www.tga.gov.au/DOCS/pdf/euguide/ich/ich37795.pdf](http://www.tga.gov.au/DOCS/pdf/euguide/ich/ich37795.pdf).
51. Australian Government Department of Health and Ageing and Therapeutic Goods Administration. Access to unapproved therapeutic goods in Australia. 2001; [www.tga.gov.au/docs/pdf/unapproved/unapp.pdf](http://www.tga.gov.au/docs/pdf/unapproved/unapp.pdf).
52. http://www.icg.org/cache/compo/276-254-1.html
53. Australian Government, National Health and Medical Research Council, Australian Research Council, Committee AV-C. National statement on ethical conduct in human research. 2007; [www.nhmrc.gov.au/publications/synopses/_files/e72.pdf](http://www.nhmrc.gov.au/publications/synopses/_files/e72.pdf).
54. Rischbieth A, Blythe D. Ethical intensive care research: development of an ethics handbook. Critical Care and Resuscitation 2005;7:310-21.

# 23 APPENDICES

23.1 Participant Information and Consent Sheet

23.2 Person Responsible Information and Consent Sheet

23.3 Patient Information Sheet (after regaining consciousness)

23.4 Product Information/Investigator Brochure

23.5 Administration and Organization Details
