# Supplementary material for: The Effect of Paracetamol on Core Body Temperature in Acute Traumatic Brain Injury: A Randomised, Controlled Clinical Trial
Source: PLoS One. 2015 Dec 17;10(12):e0144740. doi: 10.1371/journal.pone.0144740 (PMC4683067; doi:10.1371/journal.pone.0144740)
Supplement: S1 Table — (DOCX) [file pone.0144740.s003.docx]

**S1 table: Exclusion criteria related to risk of paracetamol toxicity:**

| 1. History of chronic liver disease or chronic alcohol abuse |
| --- |
| 1. Body mass index < 18 kg/m2 (or weight < 60 kg) or > 35 kg/m2 |
| 1. Serum creatinine > 200 |
| 1. Haemodynamic instability (defined as systolic blood pressure < 90 mmhg or requirement for noradrenaline or adrenaline exceeding 20mcg/minute) |
| 1. Use of hepatic enzyme inducers (except for phenytoin) |
| 1. Suspected paracetamol overdose/allergy |
