# Supplementary material for: The Effect of Paracetamol on Core Body Temperature in Acute Traumatic Brain Injury: A Randomised, Controlled Clinical Trial
Source: PLoS One. 2015 Dec 17;10(12):e0144740. doi: 10.1371/journal.pone.0144740 (PMC4683067; doi:10.1371/journal.pone.0144740)
Supplement: S2 Table — (DOCX) [file pone.0144740.s004.docx]

**S2 table: Unpublished validation study of 46 critically ill patients, evaluating the relationship between tympanic, axillary and nasopharyngeal temperature measurement, and intravesical temperature measurement.**

|  | **Mean difference (limits of agreement)**  **(^o^C)** | **Intra class coefficient**  **(95% CI)** |
| --- | --- | --- |
| Tympanic versus intravesical | 0.2 (-0.6 to 0.9) | 0.90 (0.83 0.95) |
| Axillary versus intravesical | -0.6 (-1.7 to 0.5 | 0.90 (0.83, 0.94) |
| Nasopharyngeal versus intravesical | -0.3 (-1.5 to 0.9) | 0.88 (0.79, 0.93) |
