# Supplementary material for: The Effect of Paracetamol on Core Body Temperature in Acute Traumatic Brain Injury: A Randomised, Controlled Clinical Trial
Source: PLoS One. 2015 Dec 17;10(12):e0144740. doi: 10.1371/journal.pone.0144740 (PMC4683067; doi:10.1371/journal.pone.0144740)
Supplement: S3 Table — (DOCX) [file pone.0144740.s005.docx]

**S3 table: Study Drug administration**

| Number of doses of study drug administered | Paracetamol Group  (N = 21) | Saline  Group (N = 20) | Total  (N = 41) |
| --- | --- | --- | --- |
| 0 | 1/ 21 (4.8%) | 0/ 20 (0.0%) | 1/ 41 (2.4%) |
| 5 | 0/ 21 (0.0%) | 1/ 20 (5.0%) | 1/ 41 (2.4%) |
| 8 | 1/ 21 (4.8%) | 1/ 20 (5.0%) | 2/ 41 (4.9%) |
| 10 | 0/ 21 (0.0%) | 1/ 20 (5.0%) | 1/ 41 (2.4%) |
| 12 | 2/ 21 (9.5%) | 1/ 20 (5.0%) | 3/ 41 (7.3%) |
| 15 | 1/ 21 (4.8%) | 1/ 20 (5.0%) | 2/ 41 (4.9%) |
| 16 | 0/ 21 (0.0%) | 1/ 20 (5.0%) | 1/ 41 (2.4%) |
| 17 | 4/ 21 (19.0%) | 3/ 20 (15.0%) | 7/ 41 (17.1%) |
| 18 | 12/ 21 (57.1%) | 11/ 20 (55.0%) | 23/ 41 (56.1%) |
