# Supplementary material for: The Effect of Paracetamol on Core Body Temperature in Acute Traumatic Brain Injury: A Randomised, Controlled Clinical Trial
Source: PLoS One. 2015 Dec 17;10(12):e0144740. doi: 10.1371/journal.pone.0144740 (PMC4683067; doi:10.1371/journal.pone.0144740)
Supplement: S4 Table — (DOCX) [file pone.0144740.s006.docx]

**S4 table: Mean core temperature at 24, 48 and 72 hours after randomisation**

| Mean temperature/°C (SD) | Paracetamol (N = 21) | Saline (N = 20) | Mean difference in temperature/°C (95%CI) (N = 41) | P-value |
| --- | --- | --- | --- | --- |
| 24 hours (n=39) | 37.1 (0.16) | 37.6 (0.17) | -0.45 (-0.93 to 0.03) | 0.064 |
| 48 hours (n=37) | 37.4 (0.18) | 37.7 (0.19) | -0.32 (-0.86 to 0.21) | 0.22 |
| 72 hours (n=37) | 37.6 (0.23) | 37.8 (0.23) | -0.23 (-0.89 to 0.44) | 0.49 |
