# Supplementary material for: The Effect of Paracetamol on Core Body Temperature in Acute Traumatic Brain Injury: A Randomised, Controlled Clinical Trial
Source: PLoS One. 2015 Dec 17;10(12):e0144740. doi: 10.1371/journal.pone.0144740 (PMC4683067; doi:10.1371/journal.pone.0144740)
Supplement: S5 Table — (DOCX) [file pone.0144740.s007.docx]

**S5 table: Safety outcomes**

|  | **Paracetamol (n=21)** | | | **Saline (n=20)** | | |
| --- | --- | --- | --- | --- | --- | --- |
|  | Baseline (n=21) | Day 4 (n=18) | Day 7 (n=16) | Baseline (n=20) | Day 4 (n=16) | Day 7 (n=16) |
| Alanine transferase U/L: median (IQR) | 37 (28-73) | 37 (31-44) | 59 (54-76) | 34 (21-47) | 34 (24-50) | 63 (30-94) |
| Aspartate aminotransferase U/L: median (IQR) | 58 (35-88) | 41 (32-79) | 51 (37-71) | 39 (25-69) | 37 (23-76) | 60 (30-80) |
| Bilirubin micromole/L: median (IQR) | 11 (9-19) | 10 (8-11) | 9 (8-17) | 10 (7-15) | 10 (7-11) | 9 (8-12) |
| International normalised ratio: median (IQR) | 1.2 (1.1-1.3) | 1 (1-1.1) | 1.1 (1-1.2) | 1.2 (1.1-1.2) | 1.1 (1.1-1.2) | 1.1 (1.1-1.2) |
| Abbreviations: IQR: interquartile range | | | | | | |
